# PyINETA: Open-Source Platform for INADEQUATE-JRES Integration in NMR Metabolomics

**DOI:** 10.1021/acs.analchem.4c03966

**Published:** 2024-11-20

**Authors:** Rahil Taujale, Mario Uchimiya, Chaevien S. Clendinen, Ricardo M. Borges, Christoph W. Turck, Arthur S. Edison

**Affiliations:** † Institute of Bioinformatics, University of Georgia, 120 E Green St, Athens, Georgia 30602, United States; ‡ Complex Carbohydrate Research Center, University of Georgia, 315 Riverbend Rd., Athens, Georgia 30602, United States; § The Environmental Molecular Sciences Laboratory, Pacific Northwest National Laboratory, Richland, Washington 99354, United States; ∥ Instituto de Pesquisas de Produtos Naturais, Universidade Federal do Rio de Janeiro, 21941-902 Rio de Janeiro, RJ, Brazil; ⊥ Max Planck Institute of Psychiatry, Proteomics and Biomarkers, Kraepelinstr. 2-10, 80804 Munich, Germany; # Key Laboratory of Animal Models and Human Disease Mechanisms of Yunnan Province, and KIZ/CUHK Joint Laboratory of Bioresources and Molecular Research in Common Diseases, Kunming Institute of Zoology, Chinese Academy of Sciences, Kunming 650223, China; ∇ National Resource Center for Non-human Primates, and National Research Facility for Phenotypic & Genetic Analysis of Model Animals, Kunming Institute of Zoology, Chinese Academy of Sciences, Kunming 650107, China; ○ Department of Biochemistry and Molecular Biology, University of Georgia, 120 E Green St, Athens, Georgia 30602, United States

## Abstract

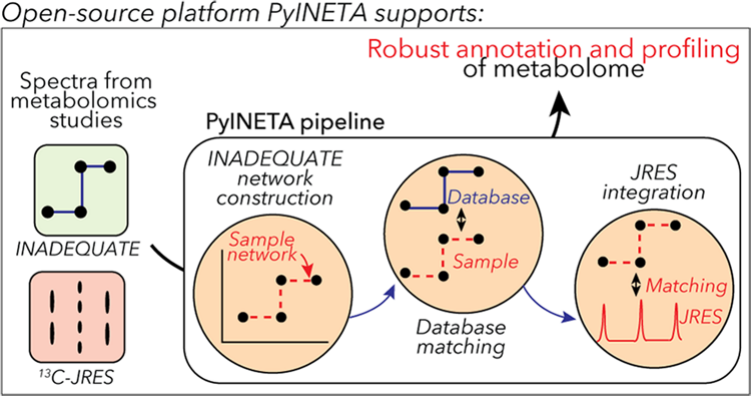

Robust annotation of compounds is a critical element in metabolomics. The ^13^C-detection NMR experiment incredible natural abundance double-quantum transfer experiment (INADEQUATE) stands out as a powerful tool for structural elucidation, but this valuable experiment is not often included in metabolomics studies. This is partly due to the lack of a community platform that provides structural information based on INADEQUATE. Also, it is often the case that a single study uses various NMR experiments synergistically to improve the quality of information or balance total NMR experiment time, but there is no public platform that can integrate the outputs of INADEQUATE with other NMR experiments. Here, we introduce PyINETA, a Python-based INADEQUATE network analysis. PyINETA is an open-source platform that provides structural information on molecules using INADEQUATE, conducts database searches using an INADEQUATE library, and integrates information on INADEQUATE and a complementary NMR experiment ^13^C *J*-resolved experiment (^13^C-JRES). ^13^C-JRES was chosen because of its ability to efficiently provide relative quantification in a study of the ^13^C-enriched samples. Those steps are carried out automatically, and PyINETA keeps track of all the pipeline parameters and outputs, ensuring the transparency of annotation in metabolomics. Our evaluation of PyINETA using a model mouse study showed that PyINETA successfully integrated INADEQUATE and ^13^C-JRES. The results showed that ^13^C-labeled amino acids that were fed to mice were transferred to different tissues and were transformed to other metabolites. The distribution of those compounds was tissue-specific, showing enrichment of specific metabolites in the liver, spleen, pancreas, muscle, or lung. PyINETA is freely available on NMRbox.

Robust annotation of compounds is a critical task in metabolomics. In NMR metabolomics, compound annotation is primarily based on chemical shifts of ^1^H, ^13^C, or both. Two-dimensional (2D) experiments increase the confidence level of annotation, providing correlations between protons or protons and carbons in molecules.^1^ Although ^1^H-detection 2D experiments have been successfully implemented in metabolomics for this purpose,^2^^13^C-detection NMR can complement ^1^H NMR and improve the quality of information.^3,4^^13^C NMR has a broader chemical shift range and fewer overlapping peaks than ^1^H NMR, which is ideal for metabolomics samples that are complex mixtures of compounds.^5^^13^C NMR can directly detect quaternary carbons, which leads to a broader coverage of carbon information in molecules. Most importantly from a perspective of structural elucidation, ^13^C NMR can directly extract the backbone structure of molecules,^6,7^ essential information in structural elucidation.

Among various ^13^C NMR experiments, INADEQUATE (incredible natural abundance double-quantum transfer experiment)^8^ stands out as a powerful tool for structural elucidation. This experiment unambiguously detects ^13^C–^13^C connectivity and extracts networks of carbons in molecules. INADEQUTE suffers from low natural abundance of ^13^C–^13^C couplings in molecules (i.e., less than 1 in every 10^4^ C–C bonds), but this experiment can benefit from isotopic enrichment and becomes applicable to metabolomics samples.^7^ Although one could apply INADEQUATE to many samples in a metabolomics study and profile the metabolome differences between samples,^7^ INADEQUATE requires a relatively long time for data collection, and this approach is not always practical especially when spectrometer time is limited. Thus, it is useful to have a profiling experiment that requires less instrument time but can be easily used with INADEQUATE. Although an obvious choice is a simple one-dimensional (1D) ^13^C experiment to profile all samples in a study, ^13^C-enriched samples have complicated peak shapes and more overlap than experiments at natural abundance ^13^C.

One approach to the problem is to use a 2D ^13^C *J*-resolved experiment (^13^C-JRES), which separates chemical shifts from coupling constants into different dimensions. A 1D projection of 2D ^13^C-JRES is free from multiplets and can be collected quickly enough for efficient profiling. The output can be statistically processed and linked to 2D spectra for a representative sample such as internal pooled sample, a mixture of aliquots of all the study samples, for annotation. It has been shown that a combination of ^13^C-JRES for profiling and INADEQUATE for annotation can achieve both reducing overall experiment time and maintaining the quality of structural information for a metabolomics study.^9^

In addition to the robust compound annotation, the benefit of introducing INADEQUATE is its suitability for computational tasks. Clendinen et al. developed INETA (INADEQUATE network analysis) that computationally constructs networks of backbone carbons in molecules using the INADEQUATE rules. In INADEQUATE, two directly bonded carbon atoms resonate at their natural frequencies along the acquisition dimension and at the sum of their frequencies along the indirect double-quantum dimension. This leads to pairs of peaks that are symmetric along a diagonal with slope 2, and these pairs of INADEQUATE peaks are then linked vertically to expand the network. INETA used the constructed networks to search an internal INADEQUATE database, which was simulated using assigned ^13^C chemical shifts and chemical structures of compounds deposited in Biological Magnetic Resonance Bank (BMRB).^10^ INETA was previously used to annotate the endo- and exometabolomes of ^13^C-enriched *Caenorhabditis elegans*.^7^

Despite the clear advantages of being able to annotate metabolites using INADEQUATE, there are several obstacles to its routine use. First, samples need to be isotopically labeled with ^13^C. Many microorganisms and plants can be uniformly enriched using a carbon source such as ^13^C-glucose at modest cost.^4^ This is more challenging for human studies but select targeted pathways using isotope tracers with ex vivo tissue slices or cell cultures are regularly studied.^11−13^ Second, access to a high-sensitivity cryogenic ^13^C NMR probe is necessary. Such probes are made commercially and can be accessed through large NMR facilities with user programs such as The National High Magnetic Field Lab (https://nationalmaglab.org/) or The Network for Advanced NMR (https://usnan.nmrhub.org/). Third, the previous software developed to perform INETA was written using Mathematica, which is not open source.^7^ Finally, the previous software was not developed to integrate INADEQUATE with the ^13^C-JRES data.

Here, we propose PyINETA, an open-source platform that can automatically integrate INADEQUATE and JRES data. In addition to the functions that were originally implemented in INETA, our new PyINETA seamlessly transfers INADEQUATE information to JRES, providing compound information for individual JRES peaks. The pipeline is run on Python, and researchers can freely implement this open-source platform to various metabolomics studies. We evaluated the applicability of PyINETA using a model mouse study, in which metabolites originating from ^13^C-labeled diet were examined.

As the number of metabolomics publications increases rapidly, the transparency of studies is becoming more critical than ever. This is especially true for compound annotation where the basis for annotation is required with significant rigor^14^ but is not always reported in publication.^15^ PyINETA is designed to report all of the annotation steps, providing a community platform that ensures the reproducibility and transparency of compound annotation in metabolomics.

## Experimental Section

### Development of PyINETA

PyINETA performs a series of tasks, including importing data, peak-picking, constructing networks, database matching for INADEQUATE spectra, and transferring the annotation information to JRES peaks. The pipeline requires two input file types: a configuration file and experimental NMR spectra. The configuration file contains all of the information relating to parameters used for the analysis. Adjustable parameters in PyINETA are summarized in Supporting Table S1. After the input files are loaded, the pipeline initializes a PyINETA class object. This object contains chemical shift values for the direct and double-quantum dimensions, along with an intensity matrix. The input spectra are those prepared by NMRPipe.^16^

Next, PyINETA defines the peaks for INADEQUATE spectra. For this step, it was pointed out that using a single intensity threshold is not sufficient to resolve peaks that are close together.^7^ To solve this issue, PyINETA uses multiple thresholds. The peak-picking algorithm begins with the highest intensity threshold (parameter “PPmax”) for collecting peaks. Then the pipeline proceeds by collecting data points at different slices until it reaches the minimum intensity threshold (“PPmin”). The number of slices between PPmax and PPmin is defined by a parameter “steps”. In every slice, PyINETA finds the local maximum for each peak that reaches the threshold and defines an area for that peak. PyINETA then combines peaks collected from all slices, where the pipeline applies threshold parameters “PPCS” and “PPDQ” to group nearby peaks. Any peaks that have their center of mass within PPCS of one another along the direct dimension and within PPDQ along the double-quantum dimension are considered to be a single peak.

Once the peaks are defined, the pipeline creates INADEQUATE networks. In INADEQUATE, pairs of directly bonded ^13^C atoms resonate at the sum of their frequencies in the vertical double-quantum dimension. This leads to pairs of peaks that are symmetric along a diagonal with slope of 2. Based on this, the defined peaks are screened. To meet the definition of horizontally aligned peaks, the difference in chemical shift between peaks in the double-quantum dimension needs to be less than a threshold (DQT). Next, for each pair of horizontal peaks, the difference between the sum of their chemical shifts in the direct dimension and the mean of their chemical shifts in the double-quantum dimension needs to be less than a threshold (SumXY). Also, for each pair, the difference in the absolute chemical shift distance from a line of *Y* = 2*X* between peaks needs to be less than a threshold (SDT). When multiple horizontal networks originate from sequential carbons in a single compound, those horizontal networks can be linked by vertically aligned peaks of a shared carbon. To meet the definition of vertically aligned peaks, two peaks need to have a chemical shift difference less than a threshold (CST) in the direct dimension.

Once networks are created, PyINETA starts database matching. In this pipeline, every peak in every network is compared with peaks in a simulated database in the PyINETA package. This simulated database is created using experimental spectra deposited in BMRB (details are in “Generation of a Simulated INADEQUATE Database section). First, when the distance between sample peaks and database peaks is less than a threshold (CSMT), peaks are considered matched. Second, when the number of chemical shift matches between database networks and sample networks is more than a threshold (NCMT), they are considered to be matched networks. Matched networks are subsequently analyzed along the double-quantum dimension. For this step, when the difference in chemical shift between database networks and sample networks is less than a threshold (DQMT), database networks are considered matched networks. Since all of the database entries that satisfy the criteria are considered matched networks in this scheme, it is possible to find multiple matches for any given network. To evaluate the resulting matches, two scores, the hit score and coverage score, are assigned to each matched network. The hit score quantifies the proportion of peaks in sample networks that matched a specific database entry, whereas the coverage score represents the proportion of peaks in a database entry that matched those in a sample network. For both hit and coverage scores, 1 is the maximum value. For JRES peaks, peak area values are calculated (Peak_Width_1D) and the presence and absence of peaks corresponding to INADEQUATE networks are defined using a threshold (Intensity_threshold_1D).

Finally, a summary file reports all of the major statistics about the number of peaks that passed every step. Results from each step are also saved as pickle files (i.e., an object serialization mechanism in Python).

The developed pipeline is installed on NMRbox.^17^ The source code is also available on GitHub (https://github.com/edisonomics/PyINETA.git) along with instructions and example data sets.

### Generation of a Simulated INADEQUATE Database

We constructed a simulated INADEQUATE database using structural information and experimental 1D ^13^C spectra deposited in BMRB,^10^ following the scheme proposed previously.^7^ It is important to note that in BMRB, there are cases where more than one chemical shift value is assigned to a single carbon when assignments are uncertain. To incorporate this validity information, we calculate an index “ambiguity score” for every compound in the PyINETA database. This index is calculated by *CA*/*CT*, where *CA* is the number of carbons that have more than one chemical shift assignments and *CT* is the total number of carbons in a molecule. The ambiguity score ranges from 0 (all carbons have no ambiguity) to 1 (all carbons are ambiguous) by definition. For example, in BMRB, all carbons for adenosine have unique chemical shifts. In this case, the ambiguity score for adenosine is 0 in the PyINET database. On the other hand, all carbons of glucose in the BMRB database have more than one chemical shift value to take care of anomers. For this case, the ambiguity score of glucose is 1 in the PyINETA database. The simulated database in this study contains 1973 entries, covering 1209 metabolites, which is available as a json file in the package. A list of compounds available in the PyINETA database, along with PyINETA ambiguity scores and BMRB entry identifications, is provided as a Supporting File. Users can set a threshold for the ambiguity score (parameter “Ambiguity”) in the configuration file, and PyINETA does not use database compounds that are more ambiguous than the threshold. When users further need to validate the BMRB information, an optional argument “-s singlePlot” is available to plot specific database compounds. Users can add new compounds to the PyINETA database using the module ‘gen_PyINETAdb.py’. Input format for this module is either NMR-STAR^18^ or tables with chemical shifts and structural information. The ambiguity information for peaks in input files is retained as ambiguity scores and reported in the final PyINETA output.

### In Vivo ^13^C Labeling and Sample Preparation

All animal experiments and protocols have been reviewed and approved by the Institutional Animal Care and Use Committee of the Max Planck Institute of Psychiatry. Three mice were fed with a diet that contained ^13^C-labeled amino acids, including eight essential and eight nonessential amino acids (Supporting Tables S2 and S3). After this feeding, the mouse tissues were collected. Dried tissue samples were resuspended in 50 μL of deuterated water and methanol (1:4 volume ratio) (Supporting Table 4), and the distribution of metabolites originating from those amino acids was analyzed. Complete procedures are described in Supporting Information page S3.

### NMR Data Collection and Processing

Data for INADEQUATE and ^13^C-JRES experiments were collected on a Bruker Avance Neo 900 MHz with a 5 mm TXO cryoprobe (Bruker), using NMR tubes with a diameter of 1.7 mm (Bruker). All of the NMR spectra were processed using NMRPipe.^16^ Detailed experimental and NMRPipe processing parameters are listed in Supporting Tables S5 and S6, respectively. Additional data processing for JRES spectra was conducted on MATLAB R2022b (MathWorks). Further details are given in Supporting Information page S4. The ALATIS numbering system^19^ was used for describing carbon numbers.

## Results and Discussion

### PyINETA Provides a Flexible Environment for INADEQUATE-JRES Integration

**Figure 1:**
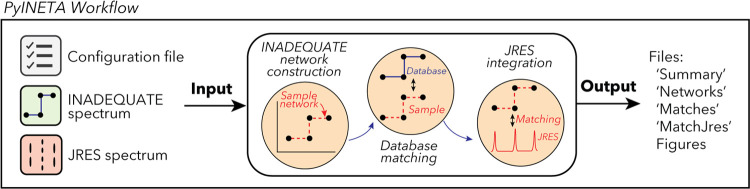
Workflow of PyINETA.

Since PyINETA uses various parameters to perform a series of tasks, we utilize a system of “configuration file” (Figure 1, left), which contains all the parameters that will be used in the pipeline (Supporting Information page S5 for an example configuration file). Users can manage and overview the pipeline using this single file. In optimizing parameters, users do not need to run the whole pipeline, and each step can be run separately by defining the “-s” option. This saves computational time.

Using parameters in a configuration file and an input INADEQUATE spectrum, the pipeline constructs INADEQUATE networks and searches for the constructed networks in an internal database (Figure 1, middle). The internal database is based on ^13^C chemical shift and chemical structure of metabolites deposited in BMRB,^7^ one of the largest databases of assigned experimental NMR data from small molecules.^10^ When users have a compound(s) of interest that are not deposited with BMRB, they can manually add those compounds to the internal database. This includes other experimental databases that provide ^13^C peaks assigned to known structures and computational data of chemical shifts from putative compounds.

Finally, as a key component, PyINETA integrates the information on INADEQUATE and ^13^C-JRES (Figure 1, middle). PyINETA reads a raw ^13^C-JRES spectrum and creates a projection. Then, peaks are picked from the projection, peaks between INADEQUATE and JRES are matched, and compound information based on INADEQUATE is transferred to JRES. We made this component optional so that users can still use this pipeline when only INADEQUATE spectra are available.

After this processing, results are reported as a set of output files (Figure 1, right). “Summary” overviews the number of peaks passed every step in INADEQUATE processing steps (Supporting Information page S10 for an example file). “Networks” provides chemical shift values for network peaks (Supporting Information page S11). “Matches” shows matched database entries, compound names, and peak connectivity information (Supporting Information page S13). The Matches file also contains confidence scores for those matches (i.e., ambiguity score, hit score, and coverage score; see Experimental Section for details), and users can evaluate the reliability of annotation. The INADEQUATE-JRES integration step is summarized in “MatchJres” (Supporting Information page S24).

In addition to those output files, PyINETA provides figures for individual networks and matched compounds for INADEQUATE (examples will follow in the next section). Similarly, PyINETA creates figures for matched peaks for INADEQUATE and JRES. This capability was implemented to enable users to further validate the results of automatic annotation. The annotation information made by this new PyINETA is consistent with that of the original study INETA^7^ (Supporting Information page S32 and Table S7).

### ^13^C-JRES Profiling Showed Clear Tissue-Specific Spectral Patterns

**Figure 2:**
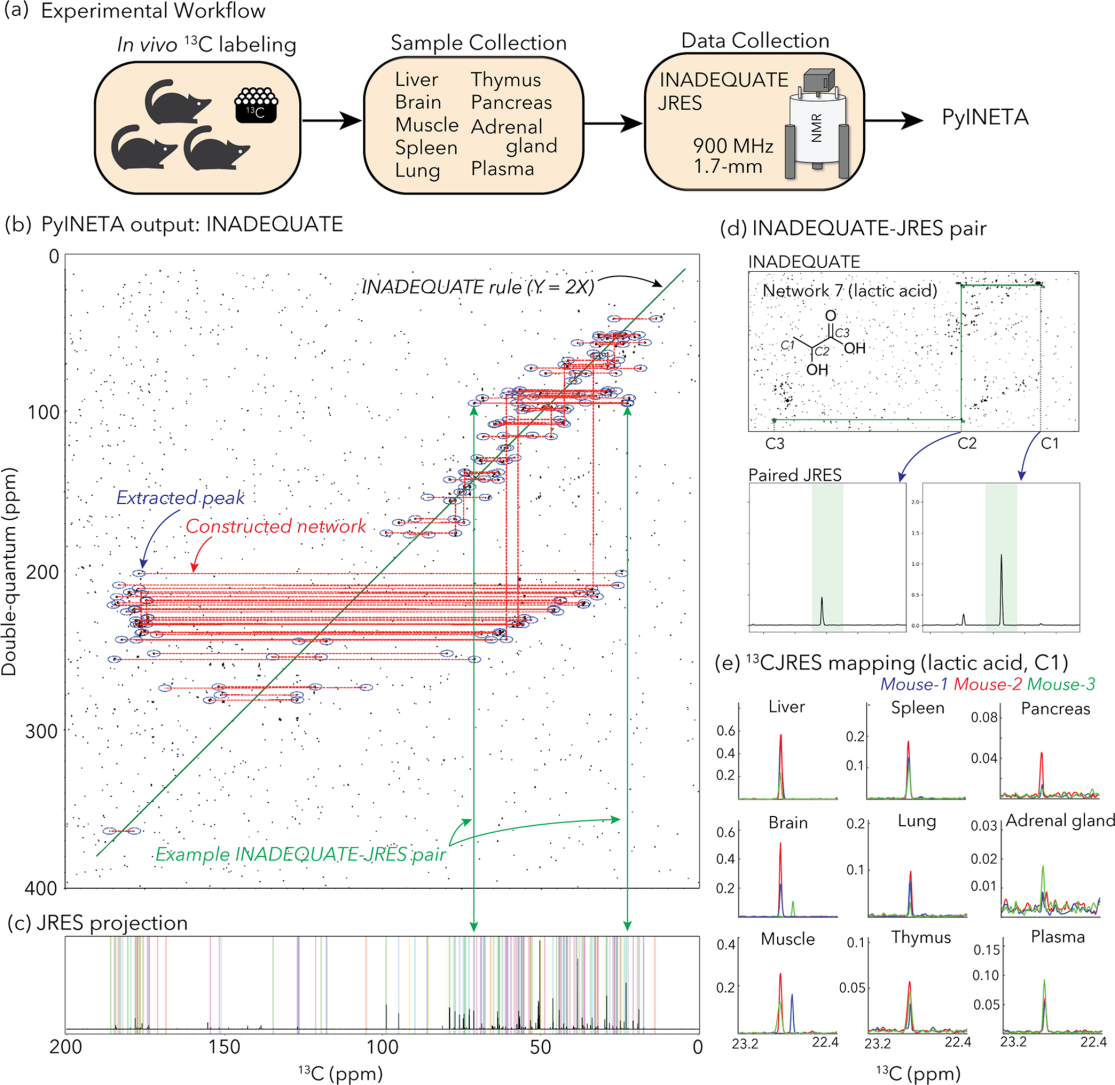
(a) Experimental workflow of this study. (b) Example of INADEQUATE networks for a mouse liver sample constructed by PyINETA. (c) JRES projection for the same sample. Peaks picked by PyINETA are highlighted in different colors. (d) INADEQUATE-JRES pair found by PyINETA. A compound name was also assigned using a database, which is lactic acid for this pair. (e) Mapping of a JRES peak in different tissues. JRES peaks for C1 for lactic acid are shown here. All of the plots for (b–d) are from the original outputs from PyINETA, with a slight graphical modification. The chemical structure was drawn using ChemDraw V23.

We applied ^13^C-JRES profiling to a mouse study where a fate of a diet was investigated (Figure 2a). Three mice were fed with a diet that contained ^13^C-labeled amino acids, including eight essential and eight nonessential amino acids (Supporting Table 3). After this feeding, mouse tissues were collected and the distribution of metabolites originating from those amino acids were analyzed in several tissues including liver, adrenal gland, lung, muscle, pancreas, plasma, brain, spleen, and thymus.

JRES spectra in those tissues were consistent among the three mice (Supporting Figure S1), and higher intensities were observed in liver samples.

### PyINETA Integrated INADEQUATE and JRES Information Automatically

Since the profiling results were consistent between mice and the liver samples had the highest intensities, we used one of the liver samples (Sample ID, 30) for the evaluation of INADEQUATE-JRES integration in PyINETA. From an INADEQUATE spectrum collected for the liver sample (Supporting Figure S2a), PyINETA constructed 67 INADEQUATE networks (Figure 2b and Supporting Information page S11 for a list of all networks). The majority (52 out of 67) of the networks had two peaks, whereas 15 networks were longer, containing 8 peaks at maximum in a network (Network 8) (Supporting Information page S11 for a complete list). Networks with just two carbons could reflect the original structure of the compound, including the case where networks are separated by heteroatoms in molecules. They could also originate from compounds with longer backbones when whole networks were not created computationally through parameter choices or factors such as signal-to-noise in the original data. We observed both situations in our output. For example, choline is a compound with a backbone of a single network (C–C) and was found in a single horizontal network of Network 44 (Supporting Figure S2b). On the other hand, lactic acid, which has a network of three carbons (C–C–C), was found in two separate horizontal networks (Networks 7 and 55) because of missing vertical connection of two horizontal networks (Supporting Figure S2c,d). Those broken networks can be manually inspected or improved by relaxing the tolerance parameter for vertical network construction (CST; Supporting Table S1).

PyINETA then searched for those 67 networks in the database, and 46 networks matched at least one candidate compound (Supporting Information page S13). The rest of the 21 networks did not have any matched compound, indicating that they are compounds that are not in BMRB (“unknown compound” hereafter).

One network can potentially match more than one compound in the database when compound structures are similar. Also, a single compound can potentially exist in more than one network, as described above. We further investigated the results using PyINETA’s function of output figures and excluded matches with less confidence due to partial structural similarity. As a result, the matched networks were those for 21 compounds (Table 1). They included amino acids (alanine, glutamine, leucine, lysine, threonine, glutamic acid, isoleucine, valine, and proline), an amino sugar (d-glucuronate), an amino alcohol (O-phosphorylethanlamine), amino sulfonic acids (hypotaurine and taurine), a pyrimidine (barbituric acid), amines (betaine, choline, ethanolamine, and putrescine), and organic acids (lactic acid and chloroacetic acid). Most of them showed no ambiguity in annotation (Table 1), except for four compounds (leucine, valine, betaine, and choline). We further investigated the original BMRB entries for those four compounds, and we confirmed that the ambiguity originates from di- or trimethyl carbons for which chemical shifts are too close to resolve. For this situation, the annotation based on INADEQUATE is still acceptable, since those residues create overlapping networks with the identical network structure and only a slight difference in chemical shifts. They also included 16 unknown compounds (Table 1).

**Table 1:** List of Compounds and Corresponding INADEQUATE Networks Detected by PyINETA for a Mouse Liver Sample

class	compound^a^	BMRB entry ID	ambiguity score	network ID in PyINETA	backbone carbons extracted^b^
amino acid	alanine	bmse000028	0	3, 41	C1–C2–C3
	glutamine	bmse000038	0	17	C1–C3
	leucine	bmse000042	0.3	32	C3–C5
	lysine	bmse000043	0	9, 10, 22	C2–C1–C3–C5
	threonine	bmse000049	0	6	C1–C2
	glutamic acid	bmse000037	0	27	C2–C4
	isoleucine	bmse000041	0	1, 2	C1–C3, C2–C4
	valine	bmse000052	0.4	4, 5	C1–C3–C2
	proline	bmse000047	0	15	C1–C3
amino sugar	d-glucuronate^c^	bmse000440	0	57	C1–C3–C6
amino alcohol	*O*-phosphoryl-ethanlamine	bmse000308	0	33	C1–C2
amino sulfonic acid	hypotaurine	bmse000452	0	25	C1–C2
	taurine	bmse000120	0	30	C1–C2
pyrimidine	barbituric acid	bmse000346	0	59	C2–C1–C3
amine	betaine	bmse000069	0.8	54	C4–C5
	choline	bmse000285	0.8	44	C4–C5
	ethanolamine	bmse000276	0	36	C1–C2
	putrescine	bmse000109	0	12	C3–C2–C1–C4
organic acid	lactic acid	bmse000208	0	7, 55	C1–C2–C3
	chloroacetic acid	bmse000367	0	38	C1–C2
	Uk-13			13	C(25.8)–C(183.0)
	Uk-16			16	C(28.0)–C(60.9)–C(182.2)
	Uk-20			20	C(31.7)-C(32.5)
	Uk-28			28	C(37.6)–C(52.6)
	Uk-34			34	C(43.7)–C(53.6)
	Uk-35			35	C(43.9)–C(174.3)–C(59.2)
	Uk-39			39	C(49.3)–C(46.8)–C(68.2)
	Uk-40			40	C(52.7)–C(61.8)
	Uk-42			42	C(55.2)–C(173.8)
	Uk-45			45	C(60.6)–C(61.5)
	Uk-46			46	C(68.9)–C(62.0)
	Uk-49			49	C(63.5)–C(78.8)
	Uk-51			51	C(65.7)–C(177.6)
	Uk-58			58	C(77.3)–C(89.7)
	Uk-62			62	C(117.6)–C(126.4)
	Uk-64			64	C(121.1)–C(151.6)

a Only a conservative list of metabolites based on the inspection of the original output is shown. The original outputs are in Supporting Information pages S11 and S13.

b For numbering of carbons, the ALATIS numbering system^19^ was used, except for unknown compounds where chemical shift values were indicated in parentheses.

c Only partial structural information was available in the BMRB entry.

Next, PyINETA analyzed a JRES spectrum collected for the same sample (Supporting Figure S2e). Among 67 INADEQUATE networks, 65 of them had JRES peaks in the corresponding regions (Figure 2c and Supporting Information page S24 for a complete list). PyINETA then transferred compound information based on INADEQUATE to JRES peaks (Figure 2d).

### PyINETA Revealed the Distribution of Metabolites Originating from a Diet in Different Tissues

**Figure 3:**
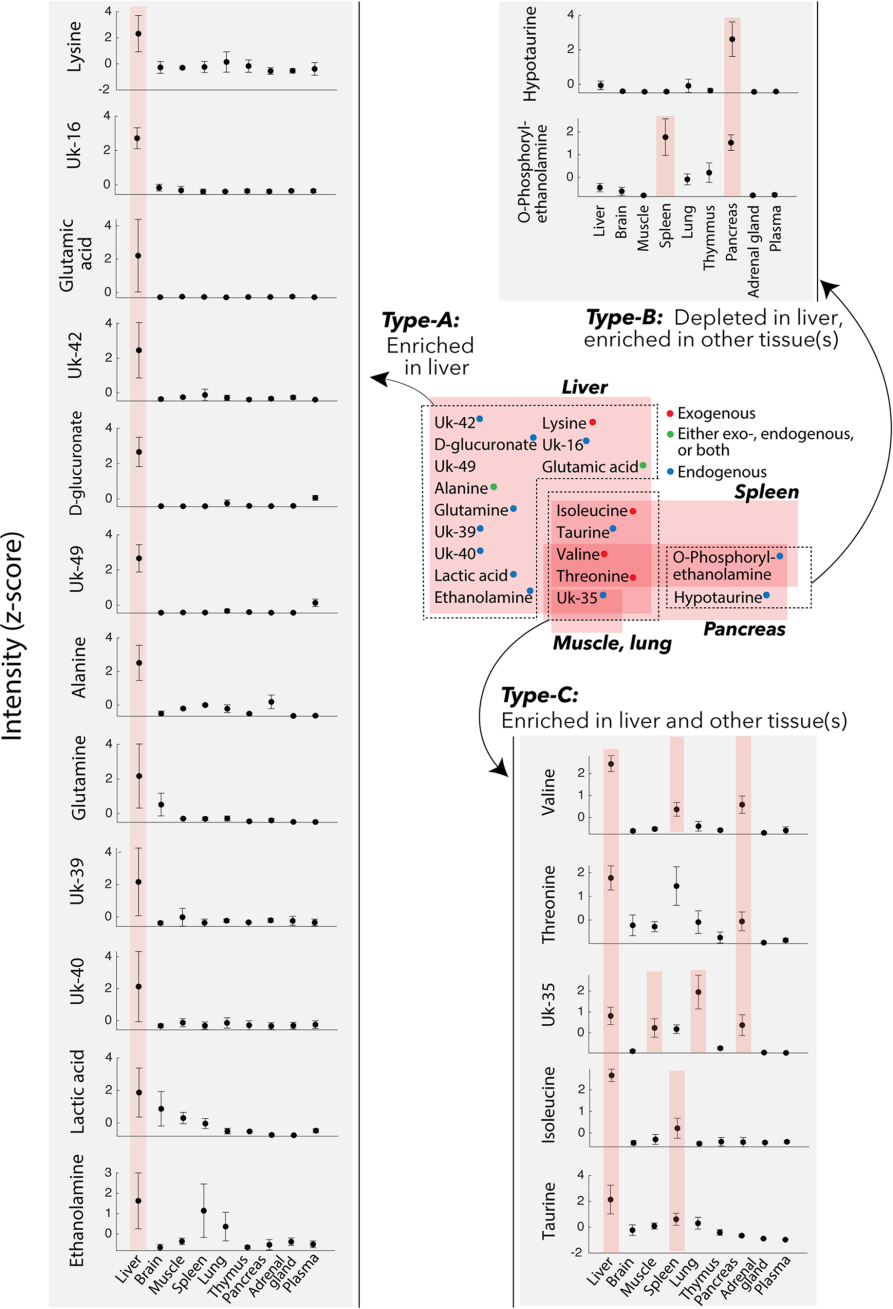
Distribution of compounds in the mouse tissues. Those are compounds originated from the ^13^C-labeled diet the mice were fed. Gray insets: peak intensities based on JRES (*z*-scored). Error bars, standard deviation (*n* = 3). When a compound in a specific tissue is enriched compared with any other tissue, it is highlighted in pink (ANOVA with multiple comparison; a complete statistical summary is in Supporting Figure S3).

Since PyINETA transformed compound information to JRES peaks, we were able to examine the distribution of a specific metabolite in different tissues based on JRES (Figure 2e). We further extended this analysis to other metabolites and examined the distribution of the metabolites in different tissues. For this analysis, we used representative JRES peaks (no overlapping peaks with a minimum intensity of 0.1), and 19 compounds are included in the following analysis. We found three different categories (Figure 3). Among the 19 compounds, 13 of them were enriched in the liver compared with other tissues (Compound Type-A) (Figure 3). Compounds in this category are amino acids (lysine, glutamic acid, alanine, and glutamine), an organic acid (lactic acid), and an amino sugar (d-glucuronate). On the other hand, two metabolites were depleted in liver but enriched in other tissue(s) (Type-B) (Figure 3); they included an amino alcohol (O-phosphorylethanlamine) in pancreas and spleen, and an amino sulfonic acid (hypotaurine) in pancreas. Finally, four compounds were enriched in both liver and other tissue(s) (muscle, spleen, lung, or pancreas; type-C; Figure 3). They are amino acids (valine, threonine, and isoleucine) and an amino sulfonic acid (taurine). Since INADEQUATE detects ^13^C–^13^C coupling in molecules that occurs less than 0.01% in natural abundance, here we interpret that the metabolites in our results originated from the ^13^C that were fed to the mice and the effects of natural abundance metabolites that were originally present in tissues are negligible.

There could be different sources of the metabolites in those three categories. Lysine, isoleucine, valine, and threonine are essential amino acids and were included in the ^13^C-labeled amino acids in the diet, suggesting that those amino acids were directly distributed from the diet to tissues (i.e., exogenous; Supporting Figure S4). Alanine and glutamic acid were also contained in the diet, but also, they are nonessential amino acids, which could be biosynthesized (i.e., endogenous), leaving a possibility that those amino acids were either exogenous, endogenous, or both (Supporting Figure S4). On the other hand, another nonessential amino acid glutamine was not included in the diet, indicating that glutamine was exclusively endogenous in this system. For metabolites other than proteinogenic amino acids (d-glucuronate, lactic acid, ethanolamine, taurine, O-phosphorylethanol amine, and hypotaurine), they are exclusively endogenous (Supporting Figure S4).

Liver plays a central role in amino acid metabolism, and this was reflected by our results. Net uptake of alanine predominantly occurs in liver,^20^ consistent with the observation of enriched alanine in liver (Type-A pattern). Alanine is further used in liver to produce other metabolites including glutamic acid,^21^ which was also in the Type-A pattern. Alanine also serves as a major precursor for gluconeogenesis which occurs in liver.^21,22^ Those transformation processes suggest that the rate of alanine input was exceeding that of transformation, resulting in the enriched alanine observed in this study. Similarly, liver is one of the dominant tissues that take up glutamine.^21,22^ On the other hand, branched-chain amino acids (BCAAs) isoleucine, valine, and threonine were not exclusively high in liver but were also abundant in other tissues (type-C). This could be due to the fact that BCAAs can escape catabolism in liver because of low activity of BCAA transferases and inefficient uptake of BCAAs in liver.^20,22,23^ Other compounds that were abundant in liver could also be a reflection of those importance in liver; lactic acid is a precursor for gluconeogenesis which occurs in liver,^24^ taurine one of the abundant amino acids with diverse physiological functions in liver,^25^ and glucuronic acid a compound that is used in glucuronidation in liver.^26^ On the contrary, hypotaurine was depleted in the liver but enriched in the pancreas. High level of hypotaurine biosynthesis occurs in pancreas in mouse.^27^

### PyINETA Also Revealed the Distribution of Unknown Compounds Originating from the Diet

PyINETA was useful even when compounds are not in the database. Out of 67 networks, 21 did not match any of the entries in BMRB. Even under that situation, we were able to track those compounds, revealing those backbone structures and distribution in different tissues (Figure 3 and Table 1). For example, Uk-16 is a compound that is not in the database, but PyINETA extracted its backbone structure and chemical shift information. Because INADEQUATE and JRES are already linked by PyINETA, we were able to trace this unknown compound using JRES and revealed the distribution in different tissues. Uk-16 was exclusively enriched in liver compared with other tissues, indicating that this is a compound that is actively processed in liver but is not in the BMRB.

In NMR metabolomics, database matching is primarily focused on peaks that match database compounds and peaks that did not match database compounds are usually not retained. On the other hand, PyINETA treats matched and unmatched networks equally and provides structural information. Since PyINETA has a capability of adding new entries to the internal database, users can make use of the obtained knowledge on unknown compounds in future studies.

Tracking metabolites using stable isotopes to understand metabolic pathway and flux has been an active field since its establishment.^11^ Despite the success and tremendous value of this approach to track targeted compounds,^12^ investigating unknown compounds in this framework is a laborious task, and effort has been made to develop untargeted approaches regardless of analytical platform.^28^ PyINETA is capable of handling unknown compounds and can contribute to tackling this challenge in this field.

### Practical Aspects of the PyINETA Pipeline in NMR Metabolomics

^13^C NMR experiments that directly detect the carbon backbones of molecules, including INADEQUATE, rely on ^13^C–^13^C couplings. Although collecting INADEQUATE spectra from natural abundance samples has been used in natural products chemistry, this approach requires a large amount of sample and is not practical in metabolomics. Because of this reason, a more practical strategy one can take is isotopic enrichment. Isotopic enrichment is an established approach in metabolomics for organisms such as bacteria,^6,29^ nematodes,^7^ terrestrial plants,^4^ and marine phytoplankton.^9^ Although there are sample types that are more challenging including human studies, tracking metabolites using isotope tracers in cell cultures or tissue slices is a regular approach.^11−13^ This field has dedicated to developing tools to analyze backbone carbons for labeled samples; they include, but not limited to, the extraction of backbone spin systems using a ^13^C–^13^C constant-time total correlation spectroscopy (CT-TOCSY) experiment,^6^ introduction of indirect covariance processing to improve backbone information in CT-TOCSY,^30^ and the development of a web-based query system to obtain compound names based on CT-TOCSY (TOCCATA, TOCSY Customized Carbon Trace Archive).^29^ They also include the introduction of INADEQUATE to metabolomics samples to extract networks of backbone carbons unambiguiously.^7^ PyINETA can be practically used for systems in metabolomics. Complementing the previous valuable tools, PyINETA provides an open-source environment for structural information and a way to integrate information of ^13^C experiments, as was seen in our model mouse experiment.

### Ensuring the Reproducibility of Compound Annotation in Metabolomics

PyINETA keeps track of all of the parameters used in the pipeline as a configuration file. Also, the results from individual steps are saved as pickle files. Those pickle files contain all the information on the PyINETA class that is required to reproduce the results. Because of this system, the annotation information from PyINETA is completely reproducible. Users can also deposit those files to databases such as Metabolomics Workbench^31^ along with original data to ensure the reproducibility of compound annotation in a study.

## Conclusions

PyINETA removes current stumbling blocks in the field of metabolomics, making the best use of ^13^C NMR and improving the transparency and reproducibility of compound annotation. In addition to the example study presented here, PyINETA can be expanded to any system that can be labeled to address specific questions in various research fields. PyINETA is installed on NMRbox^17^ and publicly available.

## Data Availability

All the raw NMR data, NMRPipe processing scripts, processed data, values for Figure 3, PyINETA output files, and MATLAB scripts used in this study are deposited to Metabolomics Workbench with Study ID ST003304.

## Supporting Information Available

Additional information for the pipeline; biological experiment; NMR data collection and processing; and results including the original spectra and statistical analysis (PDF)
List of compounds in the PyINETA database, including corresponding BMRB entry identifications and ambiguity scores (XLSX)
