# Supplementary material for: PyINETA: Open-Source Platform for INADEQUATE-JRES Integration in NMR Metabolomics
Source: Anal Chem. 2024 Nov 20;96(48):19029–37. doi: 10.1021/acs.analchem.4c03966 (PMC11618735; doi:10.1021/acs.analchem.4c03966)
Supplement: Supplementary file 1 — ac4c03966_si_001.pdf [file ac4c03966_si_001.pdf]

Supporting Information

PyINETA: Open-source platform for INADEQUATE-JRES integration in NMR metabolomics

Rahil Taujale[a,b,§], Mario Uchimiya[b,§,*], Chaevien S. Clendinen[c], Ricardo M. Borges[d], Christoph W. Turck[e,f,g], Arthur S. Edison[a,b,h,*]

[a]: *Institute of Bioinformatics, University of Georgia, 120 E Green St, Athens, GA 30602, USA*
[b]: *Complex Carbohydrate Research Center, University of Georgia, 315 Riverbend Rd., Athens, GA 30602, USA*
[c]: *The Environmental Molecular Sciences Laboratory, Pacific Northwest National Laboratory, Richland, WA, 99354 USA*
[d]: *Instituto de Pesquisas de Produtos Naturais, Universidade Federal do Rio de Janeiro, 21941-902, Rio de Janeiro, RJ, Brazil*
[e]: *Max Planck Institute of Psychiatry, Proteomics and Biomarkers, Kraepelinstr. 2-10, 80804 Munich, Germany*
[f]: *Key Laboratory of Animal Models and Human Disease Mechanisms of Yunnan Province, and KIZ/CUHK Joint Laboratory of Bioresources and Molecular Research in Common Diseases, Kunming Institute of Zoology, Chinese Academy of Sciences, Kunming, 650223, China*
[g]: *National Resource Center for Non-human Primates, and National Research Facility for Phenotypic & Genetic Analysis of Model Animals, Kunming Institute of Zoology, Chinese Academy of Sciences, Kunming, 650107, China*
[h]: *Department of Biochemistry and Molecular Biology, University of Georgia, 120 E Green St, Athens, GA 30602, USA*

[§]: *These authors contributed equally to this paper*

*: Email: Arthur S. Edison (aedison@uga.edu); Mario Uchimiya (mario.uchimiya@uga.edu)

Table of Contents

In vivo ^{13}C labeling and sample preparation S3
NMR data collection and processing S4
Example of configuration file in PyINETA S5
Example of Summary file in PyINETA S10
Example of Networks file in PyINETA S11
Example of Matches file in PyINETA S13
Example of MatchJres file in PyINETA........ S24
Evaluation of consistency of annotation between the original INETA and new PyINETA S32
Supplementary Table S1: List of adjustable parameters in PyINETA........ S33
Supplementary Table S2: Composition of the diet used in this study S34
Supplementary Table S3: Composition of amino acids in the diet S35
Supplementary Table S4: Sample list of this study S36
Supplementary Table S5: Parameters used for NMR experiments. S37
Supplementary Table S6: Parameters used for NMRPipe processing S38
Supplementary Table S7: List of metabolites found by PyINETA for endo- and exometabolites from *Caenorhabditis elegans* S39
Supplementary Figure S1: JRES projection spectra for the mouse tissues S41
Supplementary Figure S2: INADEQUATE and JRES spectra and PyINETA output figures from the mouse study S42
Supplementary Figure S3: Results of ANOVA and multiple comparison S43
Supplementary Figure S4: Possible sources of metabolites detected in this study S44
References S45

In vivo ^{13}C labeling and sample preparation

All animal experiments and protocols have been reviewed and approved by the Institutional Animal Care and Use Committee of the Max Planck Institute of Psychiatry. Three 8-week-old male C57BL/6 mice (Charles River Laboratories, Maastricht, The Netherlands) were housed under standard conditions (12-h light/dark cycle, lights on at 0600 h, room temperature 23 ± 2 °C, humidity 60%, tap water and food *ad libitum*) and fed with standard rodent diet (Harlan Laboratories, Inc., Indianapolis, IN, USA) for one week. For adaptation prior to labeling the animals were first fed an unlabeled *Ralstonia eutropha* bacterial protein-based rodent diet (Silantes GmbH, Munich, Germany) for 4 days. The food supply was then switched to ^{13}C-labeled *Ralstonia eutropha* bacterial diet (Silantes GmbH) for 14 days (Supplementary Tables S2 and S3). Following labeling the animals were sacrificed and organs and blood isolated. The partially ^{13}C-labeled animals did not show any discernible health effects compared to animals fed with a standard diet and had similar weight gains as animals fed with standard food (data not shown). Tissues were homogenized in 30 volumes of ice cold 80% methanol, homogenates were centrifuged and supernatants were dried. Dried samples were resuspended in 50 µL of deuterated water and methanol (1:4 volume ratio) (Supplementary Table 4).

NMR data collection and processing

Data were collected on a Bruker Avance Neo 900 MHz with a 5-mm TXO cryoprobe (Bruker), using NMR tubes with a diameter of 1.7 mm (Bruker). For the INADEQUATE experiment, a default pulse program with adiabatic 180° pulses (Bruker nomenclature, inadphppsp) was used. For JRES, Bruker's default pulse program (jresdcqf) was modified to implement an adiabatic 180° pulse after we verified adiabatic 180° pulse is necessary in collecting ^{13}C-JRES on our 900 MHz magnet[1]. Detailed parameter settings are in Supplementary Table S5. TopSpin 4.0.9 was used to operate the spectrometer.

All the NMR spectra were processed using NMRPipe[2]. Briefly, for both INADEQUATE and JRES, the FIDs were Fourier-transformed after applying a squared sine-bell function and a double zero-filling on both direct and indirect dimensions. JRES spectra were further tilted and symmetrized. Detailed NMRPipe processing parameters are in Supplementary Table S6. Further data processing for JRES spectra was conducted using Metabolomics Toolbox (https://github.com/edisonomics/metabolomics_toolbox) on MATLAB R2022b (MathWorks). Briefly, projection spectra were created from JRES, and they were aligned with the CCOW method (function 'guide_align1D') and normalized by the probabilistic quotient normalization (PQN) method[3] ('normalize').

All the raw data, NMRPipe processing scripts, processed data, PyINETA output files, and MATLAB scripts are available in Metabolomics Workbench with Study ID ST003304.

Example of configuration file in PyINETA

```
; This is the template for a config.ini file used with pyINETA
; Lines enclosed in "[]" indicate section headers.
; Other lines have the options and parameters required by INETA
; Lines starting with a ";" are comments and describe the options
preceding it.

[PeakPick]
Ft_File =
<path_to_the_raw_data_file>/raw_data/pyINETA_AlaRef_2023/4_inad.ft2
Data_Matrix_File =
; (optional if no Ft file)
13C_Ppm_File =
; (optional if no Ft file)
Double_Quantum_File =
; (optional if no Ft file)

Xrange_min = 0
Xrange_max = 200
; The min and max ppm values for the X-axis (13C axis)
Yrange_min = 0
Yrange_max = 400
; The min and max ppm values for the Y-axis (DQ axis)
OutImage_pick_separate = fig_1plotSeparate.eps
OutImage_pick_complete = fig_1plotAll.eps
; Filenames for output images after peak picking

Shift = No
; Yes or No | Yes to shift the spectrum - This functionality is helpful
for aligning the INADEQUATE spectrum by shifting all the peaks uniformly
across one or both axes.
Direction = Pos
; Pos or Neg | Which direction to shift the spectra in
; For eg, Pos will move a peak at 35 ppm to 40 ppm
;         Neg will move a peak at 40 ppm to 35 ppm
Shift13C = 20
; # of units to shift the spectra
; In a typical INADEQUATE spectrum, 20 units ~ 1 ppm
Full13C = 4096
; Size of the 13C dimension in units; number of columns in the data
matrix.
FullDQ = 2048

; Size of the DQ dimension in units; number of rows in the data matrix.

PPmin = 5.7e10
```

```
; Minimum intensity to select a peak
PPmax = 4e11
; Maximum intensity of a peak
steps = 10
; Number of iterations of peak picking between PPmin and PPmax
; has to be at least 2

[ClusterPoints]
PPCS = 1
; All points within this range (in ppm) along the 13C axis will be
clustered into a single point.
;
PPDQ = 2
; All points within this range (in ppm) along the DQ axis will be
clustered into a single point.

OutImage_cluster_separate = fig_2clusterCenterSeparate.eps
OutImage_cluster_complete = fig_2clusterCenterAll.eps
; Filenames for output images after clustering

[FindNetwork]
Select = all
; all or last | use "all" for most cases
; "all" iterates over all steps to find peaks
; "last" selects peaks found in the last iteration only
LevelPointsDistance = 0.5
; Typical range: 0.5 - 2
; Distance between points (in ppm) across different levels to be
considered a new peak
; Lower values results in more points.
DQT = 0.2
; Typical range: 0.5 - 2
; (threshold for DQ - diffDQ < DQT for networking)
; (Control horizontal connection to be selected for network)
SumXY = 2
; Typical range: 0.5 - 1
; (threshold for sum 2X equals Y) (Control whether Y=X1+X2)
SDT = 0.5
; Typical range: 0.2 - 0.5
; (Symmetrical/Diagonal tolerance) (Equidistant from diagonal) 0.2-0.5
best if 0.3
CST = 0.05
; (chemical shift tolerance) (Controls vertical connections), 0.07

Network_output_file = file_3Networks.txt
OutImage_network_AllNets = fig_3findNetworkAllNets.eps
```

```
[MatchDatabase]
Database_file = <path_to_pyineta>/db/InetaDB.200922.json

Ambiguity = 1
; Higher values will include more database networks, for example, 0 - will
remove all, 1 - will include all
CSMT = 1
; 0.5,2 (chemical shift match tolerance,near) Higher value will increase
the x-axis range for a match between a network and db peak
Match_tolerance = 2
; NCMT, number of peak matches, Higher values will require more number of
matching peaks to consider a database hit
DQMT = 4
; (double quantum match tolerance) Higher value will increase the y-axis
range for a match between a network and db peak
Topology_tolerance = 2
; Higher values will require x and y values to be closer to the db peak to
consider a hit
Hit_Score_threshold = 0.2
; (ratio of matched-number of peaks in db) 0-1. A value of 1 means all db
peaks need to match.A value >1 indicates that multiple candidate peaks
matched the same db peak
Coverage_Score_threshold = 0.5
; (ratio of matched-number of peaks in network) 0-1. A value of 1 requires
all network peaks to match the db peaks

Matches_list_output_file = file_4Matches.txt
Summary_file = file_Summary.txt

[Overlay1D]
1D_File_List = <path_to_1D_file>/raw_data  /file1.ft,/file2.ft,/file3.ft
; Comma separated list of 1D ft filenames
Peak_Width_1D = 0.5
; Typical range: 0.2 - 2
; When matching INADEQUATE networks with 1D spectra, this value (in ppm)
determines the peak width used to calculate peak area.
; Range of peak width calculation will be (peakPostion-Peak_Width_1D/2 to
peakPostion+Peak_Width_1D/2)
Intensity_threshold_1D = 10000
; Typical range:
; Intensity threshold for area under the curve around matched peaks to
flag as present or absent.
Match1d_output_file = file_5Match1ds.txt
OutImage_Match1d = fig_5highlight1dMatches.png
; Filename for output file with 1D matching results.
OutImageFormat1D = "png"
; Output image format: can be eps, jpeg, jpg, pdf, pgf, png, ps, raw,
```

```
rgba, svg, svgz, tif, tiff

Shift_1D = No
; Yes or No | Yes to shift all the provided 1D spectra
Direction_1D = Neg
; Pos or Neg; Which direction to shift the spectra in
; For eg, Pos will move a peak at 35 ppm is now at 40 ppm
;         Neg will move a peak at 40 ppm is now at 35 ppm
Shift_1D_val = 930
; 930 - 1.42; 780 - 1.19 ppm
; # of units to shift the spectra (650 units ~ 1 ppm for a spectra fo size
131072 units)
Full_1D = 131072
; Size of the 13C dimension in units

[OverlayJres]
Jres_File_List = /<path_to_Jres_file>/3.tilt.sym.ft2
; 40_cres.tilt.sym.ft
; Comma separated list of Jres ft filenames.

Jres_Projection_Method = max
; Can be sum,max or avg
Peak_Width_Jres = 0.5
; Typical range: 0.2 - 2
; When matching INADEQUATE networks with 1D spectra, this value (in ppm)
determines the peak width used to calculate peak area.
; Range of peak width calculation will be (peakPostion-Peak_Width_1D/2 to
peakPostion+Peak_Width_1D/2)
Intensity_threshold_Jres = 10000
; Typical range:
; Intensity threshold for area under the curve around matched peaks to
flag as present or absent.
MatchJres_output_file = file_5MatchJres.txt
OutImage_MatchJres = fig_5highlightJresMatches.png
; Filename for output file with 1D matching results.
OutImageFormatJres = png
; Output image format: can be eps, jpeg, jpg, pdf, pgf, png, ps, raw,
rgba, svg, svgz, tif, tiff

Shift_Jres = No
; Yes or No | Yes to shift all the provided 1D spectra
Direction_Jres = Neg
; Pos or Neg; Which direction to shift the spectra in
; For eg, Pos will move a peak at 35 ppm is now at 40 ppm
;         Neg will move a peak at 40 ppm is now at 35 ppm
Shift_Jres_val = 930
; 930 - 1.42; 780 - 1.19 ppm
```

```
; # of units to shift the spectra (650 units ~ 1 ppm for a spectra of size 131072 units)
Full_Jres = 131072
; Size of the 13C dimension in units
```

Example of Summary file in PyINETA

```
Summary for PyINETA run on 2024-06-07 15:17:21.483747 :
# of picked peaks in all steps: 6629
# of peaks after clustering: 2493
# of peaks retained after merging steps: 1806
# of networks found: 67
# of matches found: 46
```

Example of Networks file in PyINETA

```
Network1    [27.03 40.91],[13.72 41.01]
Network2   [38.58 56.17],[17.27 55.93]
Network3   [18.7  72.18],[53.2  71.98]
Network4   [31.73 51.09],[19.2  50.97]
Network5   [31.82 52.36],[20.57 52.45]
Network6   [68.52 90.83],[22.24 90.78]
Network7   [22.87 94.29],[22.76 93.85],[70.98 94.12]
Network8   [24.7  51.54],[42.71 69.7 ],[ 42.75 107.44],[26.95
69.7 ],[ 64.76 107.56],[ 64.36 107.25],[26.95 51.62],[23.43 50.35],[26.95
50.46]
Network9   [24.13 53.52],[29.14 53.52]
Network10  [32.59 57.04],[24.3  56.98]
Network11  [ 24.64 201.46],[176.75 201.46]
Network12  [25.69 67.17],[41.51 67.17]
Network13  [183.03 208.87],[ 25.8  209.03]
Network14  [26.42 86.41],[60.42 86.62]
Network15  [48.6  75.22],[26.53 75.15]
Network16  [182.24 243.22],[28.07 89.06],[ 60.97 243.13],[60.99 89.21]
Network17  [28.95 85.85],[56.68 85.82]
Network18  [29.03 70.84],[ 33.61 213.39],[29.04 62.72],[179.7
213.2],[33.57 62.62],[41.66 71.  ]
Network19  [176.46 233.69],[29.51 86.95],[175.96 233.  ],[57.33
86.94],[ 57.33 233.61]
Network20  [32.53 64.2 ],[31.67 64.35]
Network21  [ 32.4  216.14],[183.72 216.19]
Network22  [32.76 89.8 ],[57.01 89.9 ]
Network23  [176.93 210.88],[ 34.1  210.98]
Network24  [39.26 75.41],[40.64 80.07],[39.28 80.07],[35.96 75.41]
Network25  [57.67 93.92],[36.09 94.03]
Network26  [183.33 219.95],[ 36.39 220.  ]
Network27  [ 36.72 221.34],[184.84 221.45]
Network28  [52.6  90.24],[37.61 90.11]
Network29  [ 37.96 214.07],[176.08 214.05],[176.12 238.9 ],[ 62.97 239.03]
Network30  [49.98 88.1 ],[38.15 88.13]
Network31  [41.77 97.25],[55.36 97.25]
Network32  [56.02 98.68],[42.53 98.68]
Network33  [ 43.35 106.54],[ 63.12 106.42]
Network34  [53.69 97.62],[43.76 97.62]
Network35  [ 44.   218.17],[ 43.95 218.75],[174.37 218.7 ],[174.43
218.33],[174.34 233.4 ],[ 59.26 233.4 ]
Network36  [ 60.23 104.39],[ 44.13 104.39]
Network37  [180.62 225.68],[ 44.86 225.79]
Network38  [178.15 223.78],[178.14 224.28],[ 46.   223.94],[ 46.14 224.34]
Network39  [ 46.86 115.12],[49.38 96.25],[46.84 96.37],[ 68.23 115.23]
Network40  [ 52.76 114.37],[ 61.89 114.52]
```

```
Network41  [ 53.12 230.86],[177.71 230.86]
Network42  [ 55.28 229.4 ],[173.87 229.56],[174.04 229.21]
Network43  [177.5  233.48],[ 55.83 233.72]
Network44  [ 58.28 128.55],[ 70.17 128.58]
Network45  [ 60.68 122.31],[ 61.52 122.37]
Network46  [ 68.97 130.51],[ 62.07 130.8 ],[ 61.04 130.4 ]
Network47  [175.75 238.53],[ 63.04 238.26]
Network48  [ 74.1  146.86],[ 72.59 146.83],[ 74.1  137.65],[ 63.49 137.64]
Network49  [ 63.56 142.6 ],[ 78.81 142.35]
Network50  [ 73.62 138.44],[ 64.94 138.43]
Network51  [ 65.74 243.31],[177.61 243.39]
Network52  [ 85.82 153.46],[ 67.59 153.56]
Network53  [ 73.8  142.03],[ 67.95 141.93]
Network54  [171.19 239.93],[ 68.8  239.96]
Network55  [ 71.04 255.69],[184.53 255.64]
Network56  [ 94.87 169.46],[ 74.43 150.18],[ 75.56 150.18],[ 74.41 169.4 ]
Network57  [ 78.72 155.7 ],[ 98.79 175.98],[ 76.99 155.7 ],[ 77.01 176.11]
Network58  [ 77.39 167.15],[ 89.73 167.02]
Network59  [ 79.69 251.91],[172.08 251.96]
Network60  [ 85.51 177.14],[ 91.48 177.14]
Network61  [168.57 273.8 ],[105.15 273.37]
Network62  [126.48 244.29],[117.68 244.4 ]
Network63  [119.39 254.13],[134.66 254.13]
Network64  [121.12 272.98],[151.69 272.98]
Network65  [126.82 281.2 ],[154.51 281.4 ]
Network66  [151.08 278.03],[126.96 278.13]
Network67  [178.26 363.89],[186.07 363.89]
```

Example of Matches file in PyINETA

```
#NetworkNum ID MatchName  Solvent    AmbiguityScore Hitscore
CoverageScore  MatchedConnections UnmatchedConnections
Network1   bmse000041 L_isoleucine   D2O    0.0    0.2    1.0    CX1-CX2-
>C1-C6,
Network1   bmse000319 3_Methyl_2_oxopentanoic_acid   D2O    0.0    0.2
1.0    CX1-CX2->C5-C8,
Network1   bmse000574 3_Methyl_2_oxopentanoic_acid   D2O    0.0    0.2
1.0    CX1-CX2->C5-C8,
Network1   bmse000578 ethylmalonic_acid  D2O    0.0    0.25   1.0    CX1-
CX2->C6-C7,
Network1   bmse000866 L_isoleucine   D2O    0.0    0.2    1.0    CX1-CX2-
>C1-C6,
Network2   bmse000041 L_isoleucine   D2O    0.0    0.2    1.0    CX2-CX1-
>C7-C2,
Network2   bmse000866 L_isoleucine   D2O    0.0    0.2    1.0    CX2-CX1-
>C7-C2,
Network3   bmse000028 L_alanine  D2O    0.0    0.5    1.0    CX2-CX1->C2-
C5,
Network3   bmse000236 D_alanine  D2O    0.0    0.5    1.0    CX2-CX1->C2-
C3,
Network3   bmse000282 DL_Alanine D2O    0.0    0.5    1.0    CX2-CX1->C4-
C5,
Network3   bmse000403 Ala_Ala    D2O    0.0    0.25   1.0    CX2-CX1->C6-
C9,
Network4   bmse000052 L_valine   D2O    0.4    0.25   1.0    CX2-CX1->C7-
C5,
Network4   bmse000466 DL_beta_leucine    D2O    0.0    0.2    1.0    CX2-
CX1->C8-C4,
Network4   bmse000860 L_valine   D2O    0.4    0.25   1.0    CX2-CX1->C7-
C5,
Network5   bmse000052 L_valine   D2O    0.4    0.25   1.0    CX1-CX2->C5-
C7,
Network5   bmse000466 DL_beta_leucine    D2O    0.0    0.2    1.0    CX1-
CX2->C4-C8,
Network5   bmse000860 L_valine   D2O    0.4    0.25   1.0    CX1-CX2->C5-
C7,
Network6   bmse000049 L_threonine    D2O    0.0    0.333  1.0    CX2-CX1-
>C1-C2,
Network6   bmse000794 3_carboxypropyl_trimethyl_ammonium D2O    0.571
0.333  1.0    CX2-CX1->C5-C4,
Network6   bmse000859 L_threonine    D2O    0.0    0.333  1.0    CX2-CX1-
>C1-C2,
Network7   bmse000208 L_lactic_acid  D2O    0.0    0.5    0.667  CX3-CX1-
>C2-C3,CX3-CX3->C2-C2, CX2-CX3->?-?,
Network7   bmse000269 R_Lactate  D2O    0.0    0.5    0.667  CX3-CX1->C4-
```

```
C5,CX3-CX3->C4-C4, CX2-CX3->?-?,
Network7   bmse000979 L_lactic_acid  D2O    0.0    0.5    0.667  CX3-CX1->C2-C3,CX3-CX3->C2-C2, CX2-CX3->?-?,
Network9   bmse000043 L_lysine   D2O    0.0    0.2    1.0    CX1-CX2->C6-C7,
Network9   bmse000072 cadaverine D2O    0.0    0.25   1.0    CX1-CX2->C7-C6,
Network9   bmse000237 DL_pipecolic_acid  D2O    0.0    0.2    1.0    CX1-CX2->C4-C5,
Network9   bmse000373 isovaleric_acid    D2O    0.0    0.25   1.0    CX1-CX2->C6-C3,
Network9   bmse000411 L_Norleucine   D2O    0.0    0.2    1.0    CX1-CX2->C7-C5,
Network9   bmse000493 epsilon_caprolactone   CDCl3  0.0    0.2    1.0    CX1-CX2->C4-C3,
Network9   bmse000571 2_hydroxyhexanoic_acid D2O    0.0    0.2    1.0    CX1-CX2->C6-C4,
Network10  bmse000043 L_lysine   D2O    0.0    0.2    1.0    CX2-CX1->C6-C5,
Network10  bmse000249 methyl_4_aminobutyrate D2O    0.0    0.333  1.0    CX2-CX1->C2-C4,
Network10  bmse000372 epsilon_caprolactam    D2O    0.0    0.2    1.0    CX2-CX1->C5-C3,
Network10  bmse000429 DL_2_Aminoadipic_acid  D2O    0.0    0.2    1.0    CX2-CX1->C7-C6,
Network10  bmse000442 L_homocitrulline   D2O    0.0    0.2    1.0    CX2-CX1->C7-C9,
Network10  bmse000745 homoarginine   D2O    0.0    0.2    1.0    CX2-CX1->C7-C8,
Network11  bmse000145 N_acetyl_L_glutamine   D2O    0.286  0.2    1.0    CX1-CX2->C1-C2,
Network11  bmse000157 N_acetyl_L_alanine D2O    0.0    0.333  1.0    CX1-CX2->C9-C7,
Network11  bmse000382 N_Acetyl_L_glutamic_acid   D2O    0.0    0.2    1.0    CX1-CX2->C13-C11,
Network11  bmse000423 N_acetyl_L_aspartic_acid   D2O    0.0    0.25   1.0    CX1-CX2->C12-C11,
Network11  bmse000468 4_Acetamidobutyric_acid    D2O    0.0    0.25   1.0    CX1-CX2->C10-C9,
Network11  bmse000707 N_acetyl_histidine D2O    0.0    0.2    1.0    CX1-CX2->C14-C12,
Network11  bmse000983 O_acetyl_L_serine  D2O    1.0    0.333  1.0    CX1-CX2->C10-C9,
Network12  bmse000019 D_ornithine    D2O    0.0    0.25   1.0    CX1-CX2->C4-C5,
Network12  bmse000109 putrescine D2O    0.0    0.333  1.0    CX1-CX2->C5-C4,
```

```
Network12  bmse000116 spermidine D2O    1.0    0.2    1.0    CX1-CX2->C8-
C9,
Network12  bmse000162 L_ornithine    D2O    0.0    0.25   1.0    CX1-CX2-
>C4-C5,
Network12  bmse000195 N_alpha_acetyl_DL_ornithine    D2O    0.0    0.333
1.0    CX1-CX2->C8-C11,
Network12  bmse000249 methyl_4_aminobutyrate D2O    0.0    0.333  1.0
CX1-CX2->C2-C1,
Network12  bmse000340 gamma_Aminobutyric_acid    D2O    0.0    0.333  1.0
CX1-CX2->C4-C6,
Network12  bmse000468 4_Acetamidobutyric_acid    D2O    0.0    0.25   1.0
CX1-CX2->C5-C6,
Network12  bmse000862 putrescine D2O    0.0    0.333  1.0    CX1-CX2->C5-
C4,
Network12  bmse000871 gamma_Aminobutyric_acid    D2O    0.0    0.333  1.0
CX1-CX2->C4-C6,
Network12  bmse000897 D_ornithine    D2O    0.0    0.25   1.0    CX1-CX2-
>C4-C5,
Network12  bmse000951 spermidine D2O    1.0    0.2    1.0    CX1-CX2->C8-
C9,
Network15  bmse000047 L_proline  D2O    0.0    0.25   1.0    CX2-CX1->C6-
C7,
Network15  bmse000116 spermidine D2O    1.0    0.2    1.0    CX2-CX1->C8-
C7,
Network15  bmse000947 L_proline  D2O    0.0    0.25   1.0    CX2-CX1->C6-
C7,
Network15  bmse000951 spermidine D2O    1.0    0.2    1.0    CX2-CX1->C8-
C7,
Network17  bmse000037 L_glutamic_acid    D2O    0.0    0.25   1.0    CX2-
CX1->C4-C6,
Network17  bmse000038 L_glutamine    D2O    0.0    0.25   1.0    CX2-CX1-
>C2-C5,
Network17  bmse000194 L_arginine_L_glutamate D2O    0.0    0.2    1.0
CX2-CX1->C4-C2,
Network18  bmse000038 L_glutamine    D2O    0.0    0.5    0.667  CX5-CX3-
>C6-C5,CX5-CX2->C6-C6,CX4-CX2->C7-C6,  CX1-CX3->?-?,CX1-CX6->?-?,
Network18  bmse000565 cis_5_dodecenoic_acid  CDCl3  0.0    0.273  0.667
CX5-CX3->C3-C5,CX5-CX2->C3-C13,CX4-CX2->C14-C13,   CX1-CX3->?-?,CX1-CX6-
>?-?,
Network18  bmse000625 4_methylvaleric_acid   CDCl3  0.0    0.4    0.5
CX5-CX2->C4-C5,CX4-CX2->C8-C5, CX1-CX3->?-?,CX1-CX6->?-?,CX5-CX3->C4-?,
Network19  bmse000019 D_ornithine    D2O    0.0    0.5    0.8    CX4-CX2-
>C2-C3,CX4-CX5->C2-C2,CX5-CX5->C2-C2,CX5-CX1->C2-C1,   CX3-CX5->?-?,
Network19  bmse000029 L_arginine D2O    0.0    0.5    0.8    CX4-CX2->C2-
C5,CX4-CX5->C2-C2,CX5-CX5->C2-C2,CX5-CX1->C2-C3,   CX3-CX5->?-?,
Network19  bmse000032 L_citrulline   D2O    0.0    0.5    0.8    CX4-CX2-
>C4-C6,CX4-CX5->C4-C4,CX5-CX5->C4-C4,CX5-CX1->C4-C1,   CX3-CX5->?-?,
```

```
Network19  bmse000037 L_glutamic_acid    D2O    0.0    0.5    0.8    CX4-
CX2->C4-C6,CX4-CX5->C4-C4,CX5-CX5->C4-C4,CX5-CX1->C4-C1,   CX3-CX5->?-?,
Network19  bmse000038 L_glutamine    D2O    0.0    0.5    0.8    CX4-CX2-
>C2-C5,CX4-CX5->C2-C2,CX5-CX5->C2-C2,CX5-CX1->C2-C3,   CX3-CX5->?-?,
Network19  bmse000039 L_histidine    D2O    0.333  0.5    0.8    CX4-CX2-
>C5-C3,CX4-CX5->C5-C5,CX5-CX5->C5-C5,CX5-CX1->C5-C6,   CX3-CX5->?-?,
Network19  bmse000162 L_ornithine    D2O    0.0    0.5    0.8    CX4-CX2-
>C2-C3,CX4-CX5->C2-C2,CX5-CX5->C2-C2,CX5-CX1->C2-C7,   CX3-CX5->?-?,
Network19  bmse000185 L_glutathione_reduced  D2O    0.0    0.429  1.0
CX4-CX2->C2-C6,CX4-CX5->C2-C2,CX5-CX5->C2-C2,CX5-CX1->C2-C3,CX3-CX5->C12-
C2,
Network19  bmse000209 D_citrulline   D2O    0.0    0.5    0.8    CX4-CX2-
>C6-C11,CX4-CX5->C6-C6,CX5-CX5->C6-C6,CX5-CX1->C6-C2,  CX3-CX5->?-?,
Network19  bmse000289 S_Adenosyl_L_homocysteine  D2O    0.143  0.222  0.8
CX4-CX2->C13-C15,CX4-CX5->C13-C13,CX5-CX5->C13-C13,CX5-CX1->C13-C21,
CX3-CX5->?-?,
Network19  bmse000449 Npai_Methyl_L_histidine    D2O    0.0    0.25   0.6
CX4-CX5->C7-C7,CX5-CX5->C7-C7,CX5-CX1->C7-C11, CX4-CX2->C7-?,CX3-CX5->?-?,
Network19  bmse000711 L_arginine D2O    0.0    0.5    0.8    CX4-CX2->C2-
C5,CX4-CX5->C2-C2,CX5-CX5->C2-C2,CX5-CX1->C2-C3,   CX3-CX5->?-?,
Network19  bmse000858 L_citrulline   D2O    0.0    0.5    0.8    CX4-CX2-
>C4-C6,CX4-CX5->C4-C4,CX5-CX5->C4-C4,CX5-CX1->C4-C1,   CX3-CX5->?-?,
Network19  bmse000897 D_ornithine    D2O    0.0    0.5    0.8    CX4-CX2-
>C2-C3,CX4-CX5->C2-C2,CX5-CX5->C2-C2,CX5-CX1->C2-C1,   CX3-CX5->?-?,
Network19  bmse000952 L_glutathione_reduced  D2O    0.0    0.429  1.0
CX4-CX2->C2-C6,CX4-CX5->C2-C2,CX5-CX5->C2-C2,CX5-CX1->C2-C3,CX3-CX5->C12-
C2,
Network21  bmse000483 R_2_Pyrrolidinone_5_carboxylate    D2O    0.0
0.25   1.0    CX1-CX2->C7-C8,
Network21  bmse000878 R_2_Pyrrolidinone_5_carboxylate    D2O    0.0
0.25   1.0    CX1-CX2->C7-C8,
Network22  bmse000033 L_cystathionine    D2O    0.0    0.333  1.0    CX1-
CX2->C2-C10,
Network22  bmse000043 L_lysine   D2O    0.0    0.2    1.0    CX1-CX2->C5-
C2,
Network22  bmse000044 L_methionine   D2O    0.0    0.333  1.0    CX1-CX2-
>C5-C2,
Network22  bmse000169 L_selenomethionine D2O    0.0    0.333  1.0    CX1-
CX2->C6-C2,
Network22  bmse000291 Selenomethionine   D2O    0.0    0.333  1.0    CX1-
CX2->C7-C5,
Network22  bmse000411 L_Norleucine   D2O    0.0    0.2    1.0    CX1-CX2-
>C4-C6,
Network22  bmse000429 DL_2_Aminoadipic_acid  D2O    0.0    0.2    1.0
CX1-CX2->C6-C8,
Network22  bmse000442 L_homocitrulline   D2O    0.0    0.2    1.0    CX1-
CX2->C8-C10,
```

```
Network22  bmse000450 D_Methionine   D2O    0.0    0.333  1.0    CX1-CX2-
>C5-C6,
Network22  bmse000466 DL_beta_leucine    D2O    0.0    0.2    1.0    CX1-
CX2->C4-C5,
Network22  bmse000745 homoarginine   D2O    0.0    0.2    1.0    CX1-CX2-
>C8-C10,
Network23  bmse000493 epsilon_caprolactone   CDCl3  0.0    0.2    1.0
CX1-CX2->C8-C6,
Network24  bmse000159 beta_alanine   D2O    0.0    0.5    0.5    CX4-CX1-
>C2-C6,    CX1-CX3->C6-?,CX3-CX2->?-?,
Network24  bmse000388 Cysteamine D2O    0.0    1.0    0.5    CX4-CX1->C4-
C3,    CX1-CX3->C3-?,CX3-CX2->?-?,
Network24  bmse000967 beta_alanine   D2O    0.0    0.5    0.5    CX4-CX1-
>C2-C6,    CX1-CX3->C6-?,CX3-CX2->?-?,
Network25  bmse000452 Hypotaurine    D2O    0.0    1.0    1.0    CX1-CX2-
>C5-C6,
Network26  bmse000037 L_glutamic_acid    D2O    0.0    0.25   1.0    CX1-
CX2->C2-C7,
Network26  bmse000794 3_carboxypropyl_trimethyl_ammonium D2O    0.571
0.333  1.0    CX1-CX2->C10-C9,
Network27  bmse000037 L_glutamic_acid    D2O    0.0    0.25   1.0    CX1-
CX2->C7-C2,
Network27  bmse000183 succinic_acid  D2O    0.0    0.333  1.0    CX1-CX2-
>C6-C2,
Network27  bmse000340 gamma_Aminobutyric_acid    D2O    0.0    0.333  1.0
CX1-CX2->C5-C7,
Network27  bmse000344 4_Guanidinobutyric_acid    D2O    0.0    0.333  1.0
CX1-CX2->C7-C9,
Network27  bmse000376 N_carbamyl_L_glutamate D2O    0.0    0.25   1.0
CX1-CX2->C10-C12,
Network27  bmse000382 N_Acetyl_L_glutamic_acid   D2O    0.0    0.2    1.0
CX1-CX2->C9-C12,
Network27  bmse000468 4_Acetamidobutyric_acid    D2O    0.0    0.25   1.0
CX1-CX2->C7-C8,
Network27  bmse000871 gamma_Aminobutyric_acid    D2O    0.0    0.333  1.0
CX1-CX2->C5-C7,
Network29  bmse000030 L_asparagine   D2O    0.5    0.333  0.5    CX2-CX1-
>C6-C5,    CX2-CX3->C6-?,CX3-CX4->?-?,
Network29  bmse000041 L_isoleucine   D2O    0.0    0.2    0.5    CX3-CX4-
>C4-C3,    CX2-CX1->?-?,CX2-CX3->?-?,
Network29  bmse000049 L_threonine    D2O    0.0    0.333  0.5    CX3-CX4-
>C4-C3,    CX2-CX1->?-?,CX2-CX3->?-?,
Network29  bmse000052 L_valine   D2O    0.4    0.25   0.5    CX3-CX4->C3-
C2,    CX2-CX1->?-?,CX2-CX3->?-?,
Network29  bmse000123 trans_4_hydroxy_L_proline  D2O    0.0    0.25   0.5
CX3-CX4->C4-C1,    CX2-CX1->?-?,CX2-CX3->?-?,
Network29  bmse000148 diethyl_oxalacetate    D2O    1.0    0.2    0.5
```

```
CX3-CX4->C7-C6,    CX2-CX1->?-?,CX2-CX3->?-?,
Network29  bmse000631 O_phospho_DL_threonine D2O    0.0    0.333  0.5
CX3-CX4->C12-C10,  CX2-CX1->?-?,CX2-CX3->?-?,
Network29  bmse000741 N_methyl_L_aspartic_acid   D2O    0.4    1.0    1.0
CX2-CX1->C10-C7,CX2-CX3->C10-C8,CX3-CX4->C8-C6,
Network29  bmse000859 L_threonine    D2O    0.0    0.333  0.5    CX3-CX4-
>C4-C3,    CX2-CX1->?-?,CX2-CX3->?-?,
Network29  bmse000860 L_valine   D2O    0.4    0.25   0.5    CX3-CX4->C3-
C2,    CX2-CX1->?-?,CX2-CX3->?-?,
Network29  bmse000866 L_isoleucine   D2O    0.0    0.2    0.5    CX3-CX4-
>C4-C3,    CX2-CX1->?-?,CX2-CX3->?-?,
Network29  bmse000966 trans_4_hydroxy_L_proline  D2O    0.0    0.25   0.5
CX3-CX4->C4-C1,    CX2-CX1->?-?,CX2-CX3->?-?,
Network30  bmse000120 taurine    D2O    0.0    1.0    1.0    CX1-CX2->C2-
C7,
Network31  bmse000042 L_leucine  D2O    0.333  0.2    1.0    CX1-CX2->C5-
C2,
Network31  bmse000423 N_acetyl_L_aspartic_acid   D2O    0.0    0.25   1.0
CX1-CX2->C8-C7,
Network32  bmse000042 L_leucine  D2O    0.333  0.2    1.0    CX2-CX1->C5-
C2,
Network32  bmse000423 N_acetyl_L_aspartic_acid   D2O    0.0    0.25   1.0
CX2-CX1->C8-C7,
Network32  bmse000453 Ureidosuccinic_acid    D2O    0.0    0.333  1.0
CX2-CX1->C9-C8,
Network33  bmse000308 2_Aminoethyl_dihydrogen_phosphate  D2O    0.0    1.0
1.0    CX2-CX1->C7-C8,
Network36  bmse000276 Ethanolamine   D2O    0.0    1.0    1.0    CX2-CX1-
>C4-C3,
Network37  bmse000074 D_carnitine    D2O    0.571  0.333  1.0    CX1-CX2-
>C9-C8,
Network37  bmse000211 L_carnitine    D2O    0.0    0.333  1.0    CX1-CX2-
>C1-C2,
Network37  bmse000949 D_carnitine    D2O    0.571  0.333  1.0    CX1-CX2-
>C9-C8,
Network38  bmse000367 chloroacetic_acid  D2O    0.0    1.0    0.5    CX3-
CX3->C4-C4,CX1-CX3->C5-C4,CX1-CX1->C5-C5,  CX3-CX2->C4-?,CX4-CX4->?-?,CX2-
CX4->?-?,CX2-CX2->?-?,CX2-CX1->?-?,CX1-CX4->C5-?,
Network38  bmse000658 phenylacetylglycine    D2O    0.0    0.333  0.75
CX3-CX3->C10-C10,CX3-CX2->C10-C14,CX2-CX2->C14-C14,CX2-CX1->C14-C7,CX1-
CX3->C7-C10,CX1-CX1->C7-C7, CX4-CX4->?-?,CX2-CX4->C14-?,CX1-CX4->C7-?,
Network41  bmse000028 L_alanine  D2O    0.0    0.5    1.0    CX1-CX2->C2-
C3,
Network41  bmse000236 D_alanine  D2O    0.0    0.5    1.0    CX1-CX2->C2-
C4,
Network41  bmse000282 DL_Alanine D2O    0.0    0.5    1.0    CX1-CX2->C4-
C6,
```

```
Network43  bmse000019 D_ornithine    D2O    0.0    0.25   1.0    CX1-CX2-
>C1-C2,
Network43  bmse000038 L_glutamine    D2O    0.0    0.25   1.0    CX1-CX2-
>C3-C2,
Network43  bmse000040 L_homoserine   D2O    0.0    0.333  1.0    CX1-CX2-
>C3-C2,
Network43  bmse000042 L_leucine  D2O    0.333  0.2    1.0    CX1-CX2->C3-
C2,
Network43  bmse000044 L_methionine   D2O    0.0    0.333  1.0    CX1-CX2-
>C3-C2,
Network43  bmse000073 L_canavanine   D2O    0.0    0.333  1.0    CX1-CX2-
>C5-C2,
Network43  bmse000078 creatine   D2O    0.0    1.0    1.0    CX1-CX2->C4-
C5,
Network43  bmse000162 L_ornithine    D2O    0.0    0.25   1.0    CX1-CX2-
>C7-C2,
Network43  bmse000430 DL_homocysteine    D2O    0.0    0.333  1.0    CX1-
CX2->C8-C5,
Network43  bmse000450 D_Methionine   D2O    0.0    0.333  1.0    CX1-CX2-
>C8-C6,
Network43  bmse000897 D_ornithine    D2O    0.0    0.25   1.0    CX1-CX2-
>C1-C2,
Network43  bmse000950 creatine   D2O    0.0    1.0    1.0    CX1-CX2->C4-
C5,
Network43  bmse000983 O_acetyl_L_serine  D2O    1.0    0.333  1.0    CX1-
CX2->C8-C6,
Network44  bmse000285 Choline    D2O    0.8    1.0    1.0    CX1-CX2->C3-
C4,
Network44  bmse000953 Choline    D2O    0.8    1.0    1.0    CX1-CX2->C3-
C4,
Network47  bmse000049 L_threonine    D2O    0.0    0.333  1.0    CX2-CX1-
>C3-C4,
Network47  bmse000148 diethyl_oxalacetate    D2O    1.0    0.2    1.0
CX2-CX1->C6-C7,
Network47  bmse000631 O_phospho_DL_threonine D2O    0.0    0.333  1.0
CX2-CX1->C10-C12,
Network47  bmse000859 L_threonine    D2O    0.0    0.333  1.0    CX2-CX1-
>C3-C4,
Network48  bmse000008 D_allose   D2O    0.667  0.4    0.75   CX4-CX3->C4-
C1,CX1-CX3->C9-C1, CX2-CX1->?-?,
Network48  bmse000013 D_galactose    D2O    1.0    0.2    0.75   CX4-CX3-
>C6-C5,CX1-CX3->C5-C5, CX2-CX1->?-?,
Network48  bmse000017 D_maltose  D2O    0.917  0.2    0.75   CX4-CX3->C6-
C2,CX1-CX3->C8-C2, CX2-CX1->?-?,
Network48  bmse000018 D_mannose  D2O    1.0    0.2    0.75   CX4-CX3->C7-
C6,CX1-CX3->C6-C6, CX2-CX1->?-?,
Network48  bmse000022 D_sorbose  D2O    0.5    0.4    0.75   CX4-CX3->C8-
```

```
C10,CX1-CX3->C6-C10,   CX2-CX1->?-?,
Network48  bmse000023 D_tagatose D2O    0.833  0.4    0.75   CX4-CX3->C8-
C10,CX1-CX3->C6-C10,   CX2-CX1->?-?,
Network48  bmse000026 D_xylose   D2O    1.0    0.25   0.75   CX4-CX3->C5-
C4,CX1-CX3->C4-C4, CX2-CX1->?-?,
Network48  bmse000086 alpha_D_glucose_1_phosphate    D2O    0.0    0.4
1.0    CX4-CX3->C6-C5,CX2-CX1->C4-C5,CX1-CX3->C5-C5,
Network48  bmse000087 alpha_D_glucose_1_6_bisphosphate   D2O    0.0    0.2
0.5    CX2-CX1->C4-C5,    CX4-CX3->?-?,CX1-CX3->C5-?,
Network48  bmse000099 D_mannitol D2O    0.0    0.4    0.75   CX2-CX1->C7-
C9,CX1-CX3->C9-C3, CX4-CX3->?-?,
Network48  bmse000115 D_sorbitol D2O    1.0    0.2    0.75   CX4-CX3->C6-
C5,CX1-CX3->C5-C5, CX2-CX1->?-?,
Network48  bmse000138 D_cellobiose   D2O    1.0    0.2    0.75   CX4-CX3-
>C6-C2,CX1-CX3->C8-C2, CX2-CX1->?-?,
Network48  bmse000151 alpha_D_galactose_1_phosphate  D2O    0.0    0.4
1.0    CX4-CX3->C6-C5,CX2-CX1->C4-C5,CX1-CX3->C5-C5,
Network48  bmse000163 N_acetyl_D_glucosamine_1_phosphate D2O    0.625
0.333  0.75   CX4-CX3->C15-C10,CX1-CX3->C7-C10,  CX2-CX1->?-?,
Network48  bmse000189 D_glucosamine_6_phosphate  D2O    0.0    0.2    0.5
CX2-CX1->C3-C4,    CX4-CX3->?-?,CX1-CX3->C4-?,
Network48  bmse000233 melibiose  D2O    0.0    0.2    0.5    CX2-CX1->C9-
C7,    CX4-CX3->?-?,CX1-CX3->C7-?,
Network48  bmse000235 L_gulonolactone    D2O    0.0    0.6    1.0    CX4-
CX3->C8-C4,CX2-CX1->C2-C5,CX1-CX3->C5-C4,
Network48  bmse000865 D_tagatose D2O    0.833  0.4    0.75   CX4-CX3->C8-
C10,CX1-CX3->C6-C10,   CX2-CX1->?-?,
Network48  bmse000898 D_xylose   D2O    1.0    0.25   0.75   CX4-CX3->C5-
C4,CX1-CX3->C4-C4, CX2-CX1->?-?,
Network48  bmse000903 D_sorbose  D2O    0.5    0.4    0.75   CX4-CX3->C8-
C10,CX1-CX3->C6-C10,   CX2-CX1->?-?,
Network48  bmse000939 D_cellobiose   D2O    1.0    0.2    0.75   CX4-CX3-
>C6-C2,CX1-CX3->C8-C2, CX2-CX1->?-?,
Network48  bmse000946 D_maltose  D2O    0.917  0.2    0.75   CX4-CX3->C6-
C2,CX1-CX3->C8-C2, CX2-CX1->?-?,
Network48  bmse001006 D_galactose    D2O    1.0    0.2    0.75   CX4-CX3-
>C6-C5,CX1-CX3->C5-C5, CX2-CX1->?-?,
Network48  bmse001007 D_sorbitol D2O    1.0    0.2    0.75   CX4-CX3->C6-
C5,CX1-CX3->C5-C5, CX2-CX1->?-?,
Network48  bmse001008 D_allose   D2O    0.667  0.4    0.75   CX4-CX3->C4-
C1,CX1-CX3->C9-C1, CX2-CX1->?-?,
Network50  bmse000008 D_allose   D2O    0.667  0.2    1.0    CX2-CX1->C4-
C1,
Network50  bmse000010 D_fructose D2O    1.0    0.2    1.0    CX2-CX1->C11-
C8,
Network50  bmse000013 D_galactose    D2O    1.0    0.2    1.0    CX2-CX1-
>C6-C5,
```

```
Network50  bmse000018 D_mannose  D2O    1.0    0.2    1.0    CX2-CX1->C7-
C6,
Network50  bmse000022 D_sorbose  D2O    0.5    0.2    1.0    CX2-CX1->C8-
C10,
Network50  bmse000023 D_tagatose D2O    0.833  0.2    1.0    CX2-CX1->C8-
C10,
Network50  bmse000062 adonitol   D2O    0.0    0.25   1.0    CX2-CX1->C9-
C7,
Network50  bmse000068 L_arabitol D2O    0.0    0.25   1.0    CX2-CX1->C7-
C3,
Network50  bmse000084 gluconic_acid  D2O    0.0    1.0    1.0    CX2-CX1-
>C6-C5,
Network50  bmse000087 alpha_D_glucose_1_6_bisphosphate   D2O    0.0    0.2
1.0    CX2-CX1->C6-C5,
Network50  bmse000095 i_erythritol   D2O    0.0    0.333  1.0    CX2-CX1-
>C5-C4,
Network50  bmse000099 D_mannitol D2O    0.0    0.2    1.0    CX2-CX1->C2-
C3,
Network50  bmse000100 meso_erythritol    D2O    0.0    0.333  1.0    CX2-
CX1->C5-C4,
Network50  bmse000115 D_sorbitol D2O    1.0    0.2    1.0    CX2-CX1->C6-
C5,
Network50  bmse000121 L_threitol D2O    0.0    0.333  1.0    CX2-CX1->C5-
C4,
Network50  bmse000129 xylitol    D2O    0.0    0.25   1.0    CX2-CX1->C5-
C4,
Network50  bmse000151 alpha_D_galactose_1_phosphate  D2O    0.0    0.2
1.0    CX2-CX1->C6-C5,
Network50  bmse000184 glycerol   D2O    0.0    0.5    1.0    CX2-CX1->C6-
C1,
Network50  bmse000193 DL_alpha_glycerol_phosphate    D2O    0.0    0.5
1.0    CX2-CX1->C10-C7,
Network50  bmse000230 D_glucono_1_5_lactone  D2O    0.0    1.0    1.0
CX2-CX1->C6-C5,
Network50  bmse000235 L_gulonolactone    D2O    0.0    0.2    1.0    CX2-
CX1->C8-C4,
Network50  bmse000440 D_Glucosaminic_acid    D2O    0.0    0.2    1.0
CX2-CX1->C12-C10,
Network50  bmse000856 glycerol   D2O    0.0    0.5    1.0    CX2-CX1->C6-
C1,
Network50  bmse000865 D_tagatose D2O    0.833  0.2    1.0    CX2-CX1->C8-
C10,
Network50  bmse000869 L_arabitol D2O    0.0    0.25   1.0    CX2-CX1->C7-
C3,
Network50  bmse000903 D_sorbose  D2O    0.5    0.2    1.0    CX2-CX1->C8-
C10,
Network50  bmse001005 D_fructose D2O    1.0    0.2    1.0    CX2-CX1->C11-
```

```
C8,
Network50  bmse001006 D_galactose    D2O    1.0    0.2    1.0    CX2-CX1-
>C6-C5,
Network50  bmse001007 D_sorbitol D2O    1.0    0.2    1.0    CX2-CX1->C6-
C5,
Network50  bmse001008 D_allose   D2O    0.667  0.2    1.0    CX2-CX1->C4-
C1,
Network52  bmse000204 D_ribose_5_phosphate   D2O    1.0    0.25   1.0
CX1-CX2->C7-C6,
Network53  bmse000018 D_mannose  D2O    1.0    0.2    1.0    CX2-CX1->C7-
C6,
Network53  bmse000193 DL_alpha_glycerol_phosphate    D2O    0.0    0.5
1.0    CX2-CX1->C4-C7,
Network53  bmse000257 FMN    D2O    0.0    1.0    1.0    CX2-CX1->C18-C16,
Network54  bmse000069 betaine    D2O    0.8    1.0    1.0    CX1-CX2->C4-
C3,
Network54  bmse000948 betaine    D2O    0.8    1.0    1.0    CX1-CX2->C4-
C3,
Network55  bmse000208 L_lactic_acid  D2O    0.0    0.5    1.0    CX2-CX1-
>C1-C2,
Network55  bmse000269 R_Lactate  D2O    0.0    0.5    1.0    CX2-CX1->C6-
C4,
Network55  bmse000979 L_lactic_acid  D2O    0.0    0.5    1.0    CX2-CX1-
>C1-C2,
Network56  bmse000008 D_allose   D2O    0.667  0.2    0.75   CX4-CX2->C9-
C9,CX4-CX1->C9-C7, CX2-CX3->C9-?,
Network56  bmse000013 D_galactose    D2O    1.0    0.4    0.75   CX4-CX2-
>C2-C5,CX4-CX1->C2-C1, CX2-CX3->C5-?,
Network56  bmse000015 D_glucose  D2O    1.0    0.2    0.75   CX4-CX1->C2-
C1,    CX4-CX2->C2-?,CX2-CX3->?-?,
Network56  bmse000018 D_mannose  D2O    1.0    0.4    0.75   CX4-CX2->C3-
C6,CX4-CX1->C3-C2, CX2-CX3->C6-?,
Network56  bmse000026 D_xylose   D2O    1.0    0.25   0.75   CX4-CX1->C2-
C1,    CX4-CX2->C2-?,CX2-CX3->?-?,
Network56  bmse000086 alpha_D_glucose_1_phosphate    D2O    0.0    0.2
0.75   CX4-CX2->C2-C3,CX2-CX3->C3-C3, CX4-CX1->C2-?,
Network56  bmse000125 D_trehalose    D2O    0.833  0.2    0.75   CX4-CX2-
>C14-C5,CX4-CX1->C14-C13,  CX2-CX3->C5-?,
Network56  bmse000233 melibiose  D2O    0.0    0.2    0.5    CX2-CX3->C20-
C17,  CX4-CX2->?-?,CX4-CX1->?-?,
Network56  bmse000239 D_saccharate   D2O    0.0    0.2    0.5    CX2-CX3-
>C8-C6,    CX4-CX2->?-?,CX4-CX1->?-?,
Network56  bmse000855 D_glucose  D2O    1.0    0.2    0.75   CX4-CX1->C2-
C1,    CX4-CX2->C2-?,CX2-CX3->?-?,
Network56  bmse000876 D_trehalose    D2O    0.833  0.2    0.75   CX4-CX2-
>C14-C5,CX4-CX1->C14-C13,  CX2-CX3->C5-?,
Network56  bmse000898 D_xylose   D2O    1.0    0.25   0.75   CX4-CX1->C2-
```

```
C1,    CX4-CX2->C2-?,CX2-CX3->?-?,
Network56  bmse001006 D_galactose    D2O    1.0    0.4    0.75   CX4-CX2-
>C2-C5,CX4-CX1->C2-C1, CX2-CX3->C5-?,
Network56  bmse001008 D_allose   D2O    0.667  0.2    0.75   CX4-CX2->C9-
C9,CX4-CX1->C9-C7, CX2-CX3->C9-?,
Network57  bmse000140 D_glucuronate  D2O    0.0    1.0    1.0    CX3-CX4-
>C2-C2,CX1-CX3->C1-C2,CX2-CX4->C3-C2,
Network57  bmse000313 beta_gentiobiose   D2O    0.0    0.2    1.0    CX3-
CX4->C20-C20,CX1-CX3->C18-C20,CX2-CX4->C22-C20,
Network59  bmse000346 Barbituric_acid    D2O    0.0    0.5    1.0    CX1-
CX2->C6-C8,
Network63  bmse000039 L_histidine    D2O    0.333  0.25   1.0    CX1-CX2-
>C1-C4,
Network63  bmse000077 coumarin   CDCl3  0.0    0.25   1.0    CX1-CX2->C4-
C2,
Network63  bmse000246 L_carnosine    D2O    0.0    0.25   1.0    CX1-CX2-
>C1-C6,
Network63  bmse000448 L_Histidinol   D2O    0.0    0.25   1.0    CX1-CX2-
>C9-C7,
Network63  bmse000707 N_acetyl_histidine D2O    0.0    0.2    1.0    CX1-
CX2->C11-C9,
Network63  bmse000744 histamine  D2O    0.0    0.333  1.0    CX1-CX2->C7-
C5,
Network63  bmse000762 neostigmine    D2O    0.167  0.25   1.0    CX1-CX2-
>C11-C13,
Network65  bmse000281 Nicotinamide   D2O    0.0    0.2    1.0    CX1-CX2-
>C9-C7,
Network65  bmse000622 3_pyridinecarbonitrile D2O    0.0    0.2    1.0
CX1-CX2->C5-C4,
Network66  bmse000432 Pyridine   D2O    0.0    0.25   1.0    CX1-CX2->C6-
C4,
```

Example of MatchJres in PyINETA

```
Network1
"/Users/rtaujale/Dropbox/Projects/collaborative/13C_mouse_INETA/raw
_data/pyINETA_AlaRef_2023/3.tilt.sym.ft2=CX1[Present](27.03,40.91):1129341
600000.0;CX2[Present](13.72,41.01):688701440000.0;"

Network2
"/Users/rtaujale/Dropbox/Projects/collaborative/13C_mouse_INETA/raw_data/p
yINETA_AlaRef_2023/3.tilt.sym.ft2=CX1[Present](38.58,56.17):772994430000.0
;CX2[Present](17.27,55.93):827546400000.0;"

Network3
"/Users/rtaujale/Dropbox/Projects/collaborative/13C_mouse_INETA/raw_data/p
yINETA_AlaRef_2023/3.tilt.sym.ft2=CX1[Present](18.7,72.18):2646701000000.0
;CX2[Present](53.2,71.98):1644530800000.0;"

Network4
"/Users/rtaujale/Dropbox/Projects/collaborative/13C_mouse_INETA/raw_data/p
yINETA_AlaRef_2023/3.tilt.sym.ft2=CX1[Present](31.73,51.09):1181092500000.
0;CX2[Present](19.2,50.97):1298547300000.0;"

Network5
"/Users/rtaujale/Dropbox/Projects/collaborative/13C_mouse_INETA/raw_data/p
yINETA_AlaRef_2023/3.tilt.sym.ft2=CX1[Present](31.82,52.36):1180879700000.
0;CX2[Present](20.57,52.45):1632450800000.0;"

Network6
"/Users/rtaujale/Dropbox/Projects/collaborative/13C_mouse_INETA/raw_data/p
yINETA_AlaRef_2023/3.tilt.sym.ft2=CX1[Present](68.52,90.83):1235223000000.
0;CX2[Present](22.24,90.78):832188700000.0;"

Network7
"/Users/rtaujale/Dropbox/Projects/collaborative/13C_mouse_INETA/raw_data/p
yINETA_AlaRef_2023/3.tilt.sym.ft2=CX1[Present](22.87,94.29):5607700600000.
0;CX3[Present](70.98,94.12):2692579000000.0;"

Network8
"/Users/rtaujale/Dropbox/Projects/collaborative/13C_mouse_INETA/raw_data/p
yINETA_AlaRef_2023/3.tilt.sym.ft2=CX1[Present](24.7,51.54):1726148600000.0
;CX2[Present](42.71,69.7):1706099900000.0;CX4[Present](26.95,69.7):1141805
800000.0;CX5[Present](64.76,107.56):1144215200000.0;CX8[Present](23.43,50.
35):1606211000000.0;"

Network9
```

```
"/Users/rtaujale/Dropbox/Projects/collaborative/13C_mouse_INETA/raw_data/p
yINETA_AlaRef_2023/3.tilt.sym.ft2=CX1[Present](24.13,53.52):815090300000.0
;CX2[Present](29.14,53.52):4477700000000.0;"

Network10
"/Users/rtaujale/Dropbox/Projects/collaborative/13C_mouse_INETA/raw_data/p
yINETA_AlaRef_2023/3.tilt.sym.ft2=CX1[Present](32.59,57.04):1074266700000.
0;CX2[Present](24.3,56.98):834897440000.0;"
Network11
"/Users/rtaujale/Dropbox/Projects/collaborative/13C_mouse_INETA/raw_data/p
yINETA_AlaRef_2023/3.tilt.sym.ft2=CX1[Present](24.64,201.46):1738039100000
.0;CX2[Present](176.75,201.46):912965600000.0;"

Network12
"/Users/rtaujale/Dropbox/Projects/collaborative/13C_mouse_INETA/raw_data/p
yINETA_AlaRef_2023/3.tilt.sym.ft2=CX1[Present](25.69,67.17):993319650000.0
;CX2[Present](41.51,67.17):1120700700000.0;"

Network13
"/Users/rtaujale/Dropbox/Projects/collaborative/13C_mouse_INETA/raw_data/p
yINETA_AlaRef_2023/3.tilt.sym.ft2=CX1[Present](183.03,208.87):779128500000
.0;CX2[Present](25.8,209.03):980739600000.0;"

Network14
"/Users/rtaujale/Dropbox/Projects/collaborative/13C_mouse_INETA/raw_data/p
yINETA_AlaRef_2023/3.tilt.sym.ft2=CX1[Present](26.42,86.41):766211300000.0
;CX2[Present](60.42,86.62):740303960000.0;"

Network15
"/Users/rtaujale/Dropbox/Projects/collaborative/13C_mouse_INETA/raw_data/p
yINETA_AlaRef_2023/3.tilt.sym.ft2=CX1[Present](48.6,75.22):741234100000.0;
CX2[Present](26.53,75.15):762627160000.0;"

Network16
"/Users/rtaujale/Dropbox/Projects/collaborative/13C_mouse_INETA/raw_data/p
yINETA_AlaRef_2023/3.tilt.sym.ft2=CX1[Present](182.24,243.22):820035850000
.0;CX2[Present](28.07,89.06):1381270400000.0;CX3[Present](60.97,243.13):10
34876350000.0;"

Network17
"/Users/rtaujale/Dropbox/Projects/collaborative/13C_mouse_INETA/raw_data/p
yINETA_AlaRef_2023/3.tilt.sym.ft2=CX1[Present](28.95,85.85):4472378500000.
0;CX2[Present](56.68,85.82):7125908600000.0;"

Network18
"/Users/rtaujale/Dropbox/Projects/collaborative/13C_mouse_INETA/raw_data/p
yINETA_AlaRef_2023/3.tilt.sym.ft2=CX1[Present](29.03,70.84):4478046600000.
```

```
0;CX2[Present](33.61,213.39):2013865600000.0;CX4[Present](179.7,213.2):931
228200000.0;CX6[Present](41.66,71.0):1278682200000.0;"

Network19
"/Users/rtaujale/Dropbox/Projects/collaborative/13C_mouse_INETA/raw_data/p
yINETA_AlaRef_2023/3.tilt.sym.ft2=CX1[Present](176.46,233.69):876906100000
.0;CX2[Present](29.51,86.95):1781145800000.0;CX4[Present](57.33,86.94):129
4262500000.0;"

Network20
"/Users/rtaujale/Dropbox/Projects/collaborative/13C_mouse_INETA/raw_data/p
yINETA_AlaRef_2023/3.tilt.sym.ft2=CX1[Present](32.53,64.2):1074889560000.0
;"

Network21
"/Users/rtaujale/Dropbox/Projects/collaborative/13C_mouse_INETA/raw_data/p
yINETA_AlaRef_2023/3.tilt.sym.ft2=CX1[Present](32.4,216.14):997792700000.0
;CX2[Present](183.72,216.19):773462160000.0;"

Network22
"/Users/rtaujale/Dropbox/Projects/collaborative/13C_mouse_INETA/raw_data/p
yINETA_AlaRef_2023/3.tilt.sym.ft2=CX1[Present](32.76,89.8):863568660000.0;
CX2[Present](57.01,89.9):3025168000000.0;"

Network23
"/Users/rtaujale/Dropbox/Projects/collaborative/13C_mouse_INETA/raw_data/p
yINETA_AlaRef_2023/3.tilt.sym.ft2=CX1[Present](176.93,210.88):917193600000
.0;CX2[Present](34.1,210.98):1200629400000.0;"

Network24
"/Users/rtaujale/Dropbox/Projects/collaborative/13C_mouse_INETA/raw_data/p
yINETA_AlaRef_2023/3.tilt.sym.ft2=CX1[Present](39.26,75.41):1042545840000.
0;CX2[Present](40.64,80.07):698567500000.0;CX4[Present](35.96,75.41):10097
21000000.0;"

Network25
"/Users/rtaujale/Dropbox/Projects/collaborative/13C_mouse_INETA/raw_data/p
yINETA_AlaRef_2023/3.tilt.sym.ft2=CX1[Present](57.67,93.92):1002780160000.
0;CX2[Present](36.09,94.03):1026640250000.0;"

Network26
"/Users/rtaujale/Dropbox/Projects/collaborative/13C_mouse_INETA/raw_data/p
yINETA_AlaRef_2023/3.tilt.sym.ft2=CX1[Present](183.33,219.95):736758100000
.0;CX2[Present](36.39,220.0):1143852100000.0;"

Network27
```

```
"/Users/rtaujale/Dropbox/Projects/collaborative/13C_mouse_INETA/raw_data/p
yINETA_AlaRef_2023/3.tilt.sym.ft2=CX1[Present](36.72,221.34):2123371900000
.0;CX2[Present](184.84,221.45):806637000000.0;"

Network28
"/Users/rtaujale/Dropbox/Projects/collaborative/13C_mouse_INETA/raw_data/p
yINETA_AlaRef_2023/3.tilt.sym.ft2=CX1[Present](52.6,90.24):1226222500000.0
;CX2[Present](37.61,90.11):831301160000.0;"

Network29
"/Users/rtaujale/Dropbox/Projects/collaborative/13C_mouse_INETA/raw_data/p
yINETA_AlaRef_2023/3.tilt.sym.ft2=CX1[Present](37.96,214.07):9147714000000
.0;CX2[Present](176.08,214.05):1828480900000.0;CX4[Present](62.97,239.03):
1783091400000.0;"

Network30
"/Users/rtaujale/Dropbox/Projects/collaborative/13C_mouse_INETA/raw_data/p
yINETA_AlaRef_2023/3.tilt.sym.ft2=CX1[Present](49.98,88.1):11130542000000.
0;CX2[Present](38.15,88.13):9924519000000.0;"

Network31
"/Users/rtaujale/Dropbox/Projects/collaborative/13C_mouse_INETA/raw_data/p
yINETA_AlaRef_2023/3.tilt.sym.ft2=CX1[Present](41.77,97.25):1237302600000.
0;CX2[Present](55.36,97.25):804044200000.0;"

Network32
"/Users/rtaujale/Dropbox/Projects/collaborative/13C_mouse_INETA/raw_data/p
yINETA_AlaRef_2023/3.tilt.sym.ft2=CX1[Present](56.02,98.68):1177642100000.
0;CX2[Present](42.53,98.68):1676964700000.0;"

Network33
"/Users/rtaujale/Dropbox/Projects/collaborative/13C_mouse_INETA/raw_data/p
yINETA_AlaRef_2023/3.tilt.sym.ft2=CX1[Present](43.35,106.54):991751600000.
0;CX2[Present](63.12,106.42):1745454000000.0;"

Network34
"/Users/rtaujale/Dropbox/Projects/collaborative/13C_mouse_INETA/raw_data/p
yINETA_AlaRef_2023/3.tilt.sym.ft2=CX1[Present](53.69,97.62):763946140000.0
;CX2[Present](43.76,97.62):797534300000.0;"

Network35
"/Users/rtaujale/Dropbox/Projects/collaborative/13C_mouse_INETA/raw_data/p
yINETA_AlaRef_2023/3.tilt.sym.ft2=CX1[Present](44.0,218.17):2609389000000.
0;CX3[Present](174.37,218.7):938311750000.0;CX6[Present](59.26,233.4):8884
47960000.0;"

Network36
```

```
"/Users/rtaujale/Dropbox/Projects/collaborative/13C_mouse_INETA/raw_data/p
yINETA_AlaRef_2023/3.tilt.sym.ft2=CX1[Present](60.23,104.39):729235260000.
0;CX2[Present](44.13,104.39):2625134700000.0;"

Network37
"/Users/rtaujale/Dropbox/Projects/collaborative/13C_mouse_INETA/raw_data/p
yINETA_AlaRef_2023/3.tilt.sym.ft2=CX1[Present](180.62,225.68):736136270000
.0;CX2[Present](44.86,225.79):824767150000.0;"

Network38
"/Users/rtaujale/Dropbox/Projects/collaborative/13C_mouse_INETA/raw_data/p
yINETA_AlaRef_2023/3.tilt.sym.ft2=CX1[Present](178.15,223.78):181460910000
0.0;CX3[Present](46.0,223.94):4829206300000.0;"

Network39
"/Users/rtaujale/Dropbox/Projects/collaborative/13C_mouse_INETA/raw_data/p
yINETA_AlaRef_2023/3.tilt.sym.ft2=CX1[Present](46.86,115.12):1034964760000
.0;CX2[Present](49.38,96.25):714900200000.0;CX4[Present](68.23,115.23):722
591150000.0;"

Network40
"/Users/rtaujale/Dropbox/Projects/collaborative/13C_mouse_INETA/raw_data/p
yINETA_AlaRef_2023/3.tilt.sym.ft2=CX1[Present](52.76,114.37):1196998100000
.0;CX2[Present](61.89,114.52):1095730600000.0;"

Network41
"/Users/rtaujale/Dropbox/Projects/collaborative/13C_mouse_INETA/raw_data/p
yINETA_AlaRef_2023/3.tilt.sym.ft2=CX1[Present](53.12,230.86):1627207500000
.0;CX2[Present](177.71,230.86):915893500000.0;"

Network42
"/Users/rtaujale/Dropbox/Projects/collaborative/13C_mouse_INETA/raw_data/p
yINETA_AlaRef_2023/3.tilt.sym.ft2=CX1[Present](55.28,229.4):813073200000.0
;CX2[Present](173.87,229.56):1173121000000.0;"

Network43
"/Users/rtaujale/Dropbox/Projects/collaborative/13C_mouse_INETA/raw_data/p
yINETA_AlaRef_2023/3.tilt.sym.ft2=CX1[Present](177.5,233.48):787060900000.
0;CX2[Present](55.83,233.72):1235917300000.0;"

Network44
"/Users/rtaujale/Dropbox/Projects/collaborative/13C_mouse_INETA/raw_data/p
yINETA_AlaRef_2023/3.tilt.sym.ft2=CX1[Present](58.28,128.55):702064950000.
0;CX2[Present](70.17,128.58):787518400000.0;"

Network45
```

```
"/Users/rtaujale/Dropbox/Projects/collaborative/13C_mouse_INETA/raw_data/p
yINETA_AlaRef_2023/3.tilt.sym.ft2=CX1[Present](60.68,122.31):772474140000.
0;"

Network46
"/Users/rtaujale/Dropbox/Projects/collaborative/13C_mouse_INETA/raw_data/p
yINETA_AlaRef_2023/3.tilt.sym.ft2=CX1[Present](68.97,130.51):958458230000.
0;CX2[Present](62.07,130.8):1200856200000.0;CX3[Present](61.04,130.4):1019
011200000.0;"

Network47
"/Users/rtaujale/Dropbox/Projects/collaborative/13C_mouse_INETA/raw_data/p
yINETA_AlaRef_2023/3.tilt.sym.ft2=CX1[Present](175.75,238.53):780957060000
.0;CX2[Present](63.04,238.26):1752197800000.0;"

Network48
"/Users/rtaujale/Dropbox/Projects/collaborative/13C_mouse_INETA/raw_data/p
yINETA_AlaRef_2023/3.tilt.sym.ft2=CX1[Present](74.1,146.86):2497356800000.
0;CX2[Present](72.59,146.83):4374824700000.0;CX4[Present](63.49,137.64):36
40010000000.0;"

Network49
"/Users/rtaujale/Dropbox/Projects/collaborative/13C_mouse_INETA/raw_data/p
yINETA_AlaRef_2023/3.tilt.sym.ft2=CX1[Present](63.56,142.6):3657581700000.
0;CX2[Present](78.81,142.35):5037370000000.0;"

Network50
"/Users/rtaujale/Dropbox/Projects/collaborative/13C_mouse_INETA/raw_data/p
yINETA_AlaRef_2023/3.tilt.sym.ft2=CX1[Present](73.62,138.44):806795100000.
0;CX2[Present](64.94,138.43):1153838100000.0;"

Network51
"/Users/rtaujale/Dropbox/Projects/collaborative/13C_mouse_INETA/raw_data/p
yINETA_AlaRef_2023/3.tilt.sym.ft2=CX1[Present](65.74,243.31):759493750000.
0;CX2[Present](177.61,243.39):912987100000.0;"

Network52
"/Users/rtaujale/Dropbox/Projects/collaborative/13C_mouse_INETA/raw_data/p
yINETA_AlaRef_2023/3.tilt.sym.ft2=CX1[Present](85.82,153.46):718851300000.
0;CX2[Present](67.59,153.56):808582050000.0;"

Network53
"/Users/rtaujale/Dropbox/Projects/collaborative/13C_mouse_INETA/raw_data/p
yINETA_AlaRef_2023/3.tilt.sym.ft2=CX1[Present](73.8,142.03):982890840000.0
;CX2[Present](67.95,141.93):969492270000.0;"

Network54
```

```
"/Users/rtaujale/Dropbox/Projects/collaborative/13C_mouse_INETA/raw_data/p
yINETA_AlaRef_2023/3.tilt.sym.ft2=CX1[Present](171.19,239.93):722172400000
.0;CX2[Present](68.8,239.96):978746540000.0;"

Network55
"/Users/rtaujale/Dropbox/Projects/collaborative/13C_mouse_INETA/raw_data/p
yINETA_AlaRef_2023/3.tilt.sym.ft2=CX1[Present](71.04,255.69):2677792300000
.0;CX2[Present](184.53,255.64):1155736800000.0;"

Network56
"/Users/rtaujale/Dropbox/Projects/collaborative/13C_mouse_INETA/raw_data/p
yINETA_AlaRef_2023/3.tilt.sym.ft2=CX1[Present](94.87,169.46):2254432000000
.0;CX2[Present](74.43,150.18):1827269200000.0;CX3[Present](75.56,150.18):2
142712600000.0;"

Network57
"/Users/rtaujale/Dropbox/Projects/collaborative/13C_mouse_INETA/raw_data/p
yINETA_AlaRef_2023/3.tilt.sym.ft2=CX1[Present](78.72,155.7):5029263600000.
0;CX2[Present](98.79,175.98):3195300700000.0;CX3[Present](76.99,155.7):250
6849500000.0;"

Network58
"/Users/rtaujale/Dropbox/Projects/collaborative/13C_mouse_INETA/raw_data/p
yINETA_AlaRef_2023/3.tilt.sym.ft2=CX1[Present](77.39,167.15):853489000000.
0;CX2[Present](89.73,167.02):741308200000.0;"

Network59
"/Users/rtaujale/Dropbox/Projects/collaborative/13C_mouse_INETA/raw_data/p
yINETA_AlaRef_2023/3.tilt.sym.ft2=CX1[Present](79.69,251.91):721369000000.
0;CX2[Present](172.08,251.96):731503600000.0;"

Network60
"/Users/rtaujale/Dropbox/Projects/collaborative/13C_mouse_INETA/raw_data/p
yINETA_AlaRef_2023/3.tilt.sym.ft2=CX1[Present](85.51,177.14):669664540000.
0;CX2[Present](91.48,177.14):719250200000.0;"

Network61
"/Users/rtaujale/Dropbox/Projects/collaborative/13C_mouse_INETA/raw_data/p
yINETA_AlaRef_2023/3.tilt.sym.ft2=CX1[Present](168.57,273.8):726629500000.
0;CX2[Present](105.15,273.37):786833700000.0;"

Network62
"/Users/rtaujale/Dropbox/Projects/collaborative/13C_mouse_INETA/raw_data/p
yINETA_AlaRef_2023/3.tilt.sym.ft2=CX1[Present](126.48,244.29):787265360000
.0;CX2[Present](117.68,244.4):738509000000.0;"

Network63
```

```
"/Users/rtaujale/Dropbox/Projects/collaborative/13C_mouse_INETA/raw_data/p
yINETA_AlaRef_2023/3.tilt.sym.ft2=CX1[Present](119.39,254.13):741678700000
.0;CX2[Present](134.66,254.13):770011300000.0;"

Network64
"/Users/rtaujale/Dropbox/Projects/collaborative/13C_mouse_INETA/raw_data/p
yINETA_AlaRef_2023/3.tilt.sym.ft2=CX1[Present](121.12,272.98):734866500000
.0;CX2[Present](151.69,272.98):850254230000.0;"

Network65
"/Users/rtaujale/Dropbox/Projects/collaborative/13C_mouse_INETA/raw_data/p
yINETA_AlaRef_2023/3.tilt.sym.ft2=CX1[Present](126.82,281.2):1024876700000
.0;CX2[Present](154.51,281.4):980925200000.0;"

Network66
"/Users/rtaujale/Dropbox/Projects/collaborative/13C_mouse_INETA/raw_data/p
yINETA_AlaRef_2023/3.tilt.sym.ft2=CX1[Present](151.08,278.03):750467500000
.0;CX2[Present](126.96,278.13):1001294860000.0;"

Network67
"/Users/rtaujale/Dropbox/Projects/collaborative/13C_mouse_INETA/raw_data/p
yINETA_AlaRef_2023/3.tilt.sym.ft2=CX1[Present](178.26,363.89):185129360000
0.0;CX2[Present](186.07,363.89):793620840000.0;"
```

Evaluation of the consistency of annotation between the original INETA and PyINETA

We evaluated the performance of our new Python pipeline. For this evaluation, we used the same INADEQUATE spectra that were used in the original study of INETA[5] as input files for PyINETA. They are INADEQAUATE spectra collected for endo- and exometabolites of ^{13}C-labeled *Caenorhabditis elegans*. The results were consistent (Supplementary Table 7); out of 29 metabolites annotated in the previous study, 26 were found in the new pipeline. They covered a range of compounds, including amino acids, an amino acid derivative (creatine), organic acids, a fatty acid (palmitic acid), a polyamine (putrescine), a pyrimidine derivative (uracil), and sugar derivatives. Three of them that were not included in our results are *N*-acetylglycine, stearic acid, and glutathione oxidized.

Supplementary Table S1. List of adjustable parameters in PyINETA. Those parameters are contained in each configuration file.

Parameter	Function	Value used for the mouse study	Note
For INADEQUATE			
Peak picking			
PPmin	Peak intensity minimum threshold	5.7e10	Clendinen et al. (2015)
PPmax	Peak intensity minimum threshold	4e11	Clendinen et al. (2015)
steps	Number of iterations	10	This study
PPCS	Chemical shift threshold for clustering	1	Clendinen et al. (2015)
PPDQ	Double quantum threshold for clustering	2	Clendinen et al. (2015)
Network finding			
DQT	Double quantum tolerance	0.2 ppm	Clendinen et al. (2015)
SumXY	Sum tolerance	2	This study
SDT	Symmetrical/diagonal tolerance	0.5 ppm	Clendinen et al. (2015)
CST	Chemical shift tolerance for vertical connection	0.05 ppm	Clendinen et al. (2015)
Database matching			
Ambiguity	Ambiguity allowance	1	Clendinen et al. (2015)
CSMT	Chemical shift match tolerance	1 ppm	Clendinen et al. (2015)
NCMT	Peak match tolerance	2	Clendinen et al. (2015)
DQMT	Double quantum threshold	4	Clendinen et al. (2015)
For JRES			
Peak_Width_1D	JRES peak width to be used to calculate peak area	0.5 ppm	This study
Intensity_threshold_1D	Intensity threshold to define presence or absence of peak	10,000	This study

Supplementary Table S2. Composition of the diet that was fed to the three mice in this study. For details on the composition of bacterial ^{13}C protein hydrolysate, see Supplementary Table S3.

**Component**	**g/kg**
Sucrose	350.0
Bacterial ^{13}C protein hydrolysate	200.0
Maltodextrin	130.0
Corn Starch	100.0
Soybean Oil	80.0
Cellulose	72.2
Mineral Mix, AIN-93M-MX (94049)	45.0
Vitamin Mix, AIN-93-VX (94047)	14.0
L-Cystine	3.0
Calcium Phosphate, dibasic	3.0
Choline Bitartrate	2.5
Ferric Citrate	0.25
THBQ, antioxidant	0.024

Supplementary Table S3. Amino acid composition of the hydrolysate that was fed to the three mice in this study (See also for Supplementary Table S2). The data are from a product sheet from Silantes. *: essential amino acid

**Amino acid**	**Percent**
Asp	20.56
Thr*	3.70
Ser	4.10
Glu	11.10
Gly	11.10
Ala	14.8
Val*	2.98
Met*	1.78
Ileu*	2.10
Leu*	5.26
Tyr	1.80
Phe*	2.40
His*	9.65
Lys*	3.38
Arg	2.30
Pro	2.99

Supplementary Table S4. Amount of the samples used for the mouse samples in this study. The weight is based on dry weight.

**Tissue**	**Mouse ID**	**Weight or volume**
Liver	1	1092 mg
	2	998.9 mg
	3	1000 mg
Adrenal Gland	1	32.5 mg
	2	22.3 mg
	3	36.6 mg
Lung – Only 75% of lung tissue	1	203.1 mg
	2	353.9 mg
	3	171.2 mg
Muscle – Only 50% of muscle tissue	1	121.1 mg
	2	137.6 mg
	3	82.7 mg
Pancreas	1	162.4 mg
	2	152.6 mg
	3	165.9 mg
Plasma	1	50 µL
	2	50 µL
	3	50 µL
Brain	1	226.5 mg
	2	204.3 mg
	3	226.5 mg
Spleen	1	155.7 mg
	2	154.8 mg
	3	184.3 mg
Thymus	1	59.83 mg
	2	105.6 mg
	3	68.3 mg

Supplementary Table S5. Parameters used for NMR experiments. SW, spectral width; TD, size of FID; NS, number of scans.

Figure	Experiment	Pulse program	SW (ppm)		TD		NS	Note
			F2	F1	F2	F1		
2	INADEAUATE	inadphppsp	214	433	4096	2048	16	
2, 3	JRES	jresdcqf	214	0.66	32768	50	64	The original uniform 180° pulse was replaced with an adiabatic pulse

Supplementary Table S6. Summary of NMRPipe processing parameters used for INADEQUATE and JRES experiments. SP: adjustable sine window function, ZF: zero filling. Original NMRPipe scripts are also available in Metabolomics Workbench.

Figure	Experiment	SP<br>F2 and F1	ZF<br>F2 and F1	Baseline correction<br>F2 and F1	Note
2	INADEQUATE	-off 0.0 -end 0.95 -pow 2	-auto	MED	–
2, 3	JRES	-off 0.0 -end 0.98 -pow 2	-auto	–	Magnitude calculation (MC) was additionally used; spectra were further tilted and symmetrized

Supplementary Table S7. List of metabolites found by PyINETA for endo- and exometabolites from *Caenorhabditis elegans*. See also Experimental Section for the details on the confidence scores.

Compound name	Ambiguity score	Hit score	Coverage Score	Type
Endometabolites				
Proline	0	0.25	1	Found in both the previous and this studies
Valeric acid	0	0.25	1	Found in both the previous and this studies
Glucose 1,6 bisphosphate	0	0.2	1	Found in both the previous and this studies
Histidine	0.333	0.25	1	Found in both the previous and this studies
Threonine	0	0.333	1	Found in both the previous and this studies
Arabitol	0	0.25	1	Found in both the previous and this studies
Alanine	0	0.5	1	Found in both the previous and this studies
Isoleucine	0	0.2	1	Found in both the previous and this studies
Propionic acid	0	0.5	1	Found in both the previous and this studies
3-hydroxybutyrate	0	0.333	1	Found in both the previous and this studies
Lactic acid	0	0.5	1	Found in both the previous and this studies
Valine	0.4	0.25	1	Found in both the previous and this studies
Palmitic acid	0	0.2	0.625	Found in both the previous and this studies
Exometabolites				
Isoleucine	0	0.2	1	Found in both the previous and this studies
Alanine	0	0.5	1	Found in both the previous and this studies
Threonine	0	0.333	1	Found in both the previous and this studies
Glycine	0	1	1	Found in both the previous and this studies
Proline	0	0.25	1	Found in both the previous and this studies
Ornithine	0	0.25	1	Found in both the previous and this studies
Serine	0	0.5	1	Found in both the previous and this studies

Glucose 1,6 bisphosphate	0	0.2	1	Found in both the previous and this studies
Lysine	0	0.2	1	Found in both the previous and this studies
Glutamine	0	0.25	1	Found in both the previous and this studies
Lactic acid	0	0.5	1	Found in both the previous and this studies
Glucoronate	0	0.5	0.75	Found in both the previous and this studies
Putrescine	0	0.333	1	Found in both the previous and this studies
Acetic acid	0	1	1	Found in both the previous and this studies
Arabitol	0	0.25	1	Found in both the previous and this studies
Creatine	0	1	1	Found in both the previous and this studies
Valine	0.4	0.25	1	Found in both the previous and this studies
Allantoin	0	1	0.667	Found in both the previous and this studies
Propionic acid	0	0.5	1	Found in both the previous and this studies
Succinic acid	0	0.333	1	Found in both the previous and this studies
Methionine	0	0.333	0.5	Found in both the previous and this studies
Uracil	0	0.5	1	Found in both the previous and this studies

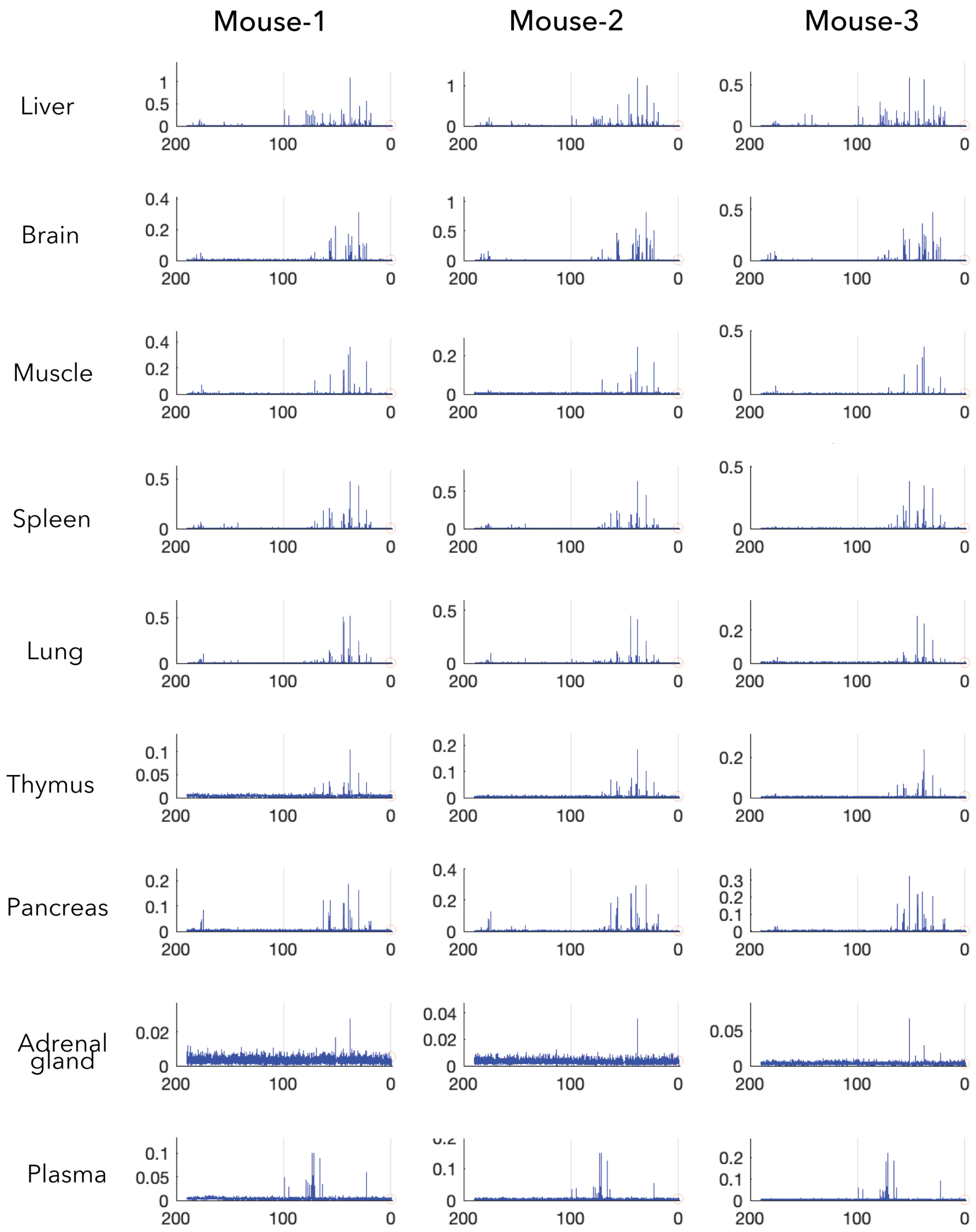

Supplementary Figure S1 JRES projection spectra for the mouse tissues. Solvent MeOH peaks around 50 ppm are removed and not shown.

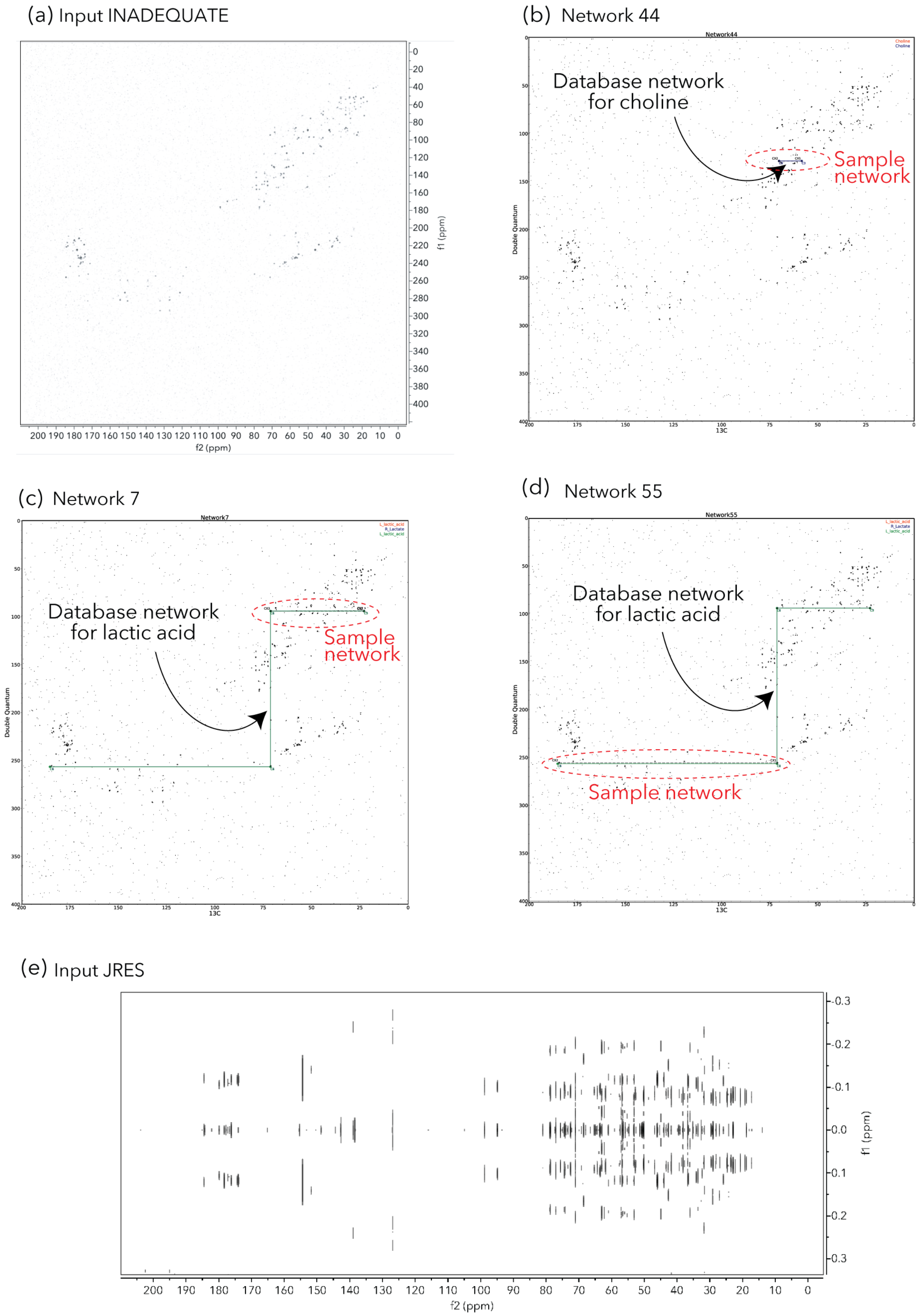

Supplementary Figure S2 (a) Input INADEQUATE spectrum for the liver sample; (b) Network 44 of PyINETA; (c) Network 7; (d) Network 55; (e) Input JRES spectrum for the liver sample. For a and e, the plots were produced by MestReNova v14.3.0. For b, c, and d, the plots are the original outputs from PyINETA, with a slight graphical modification.

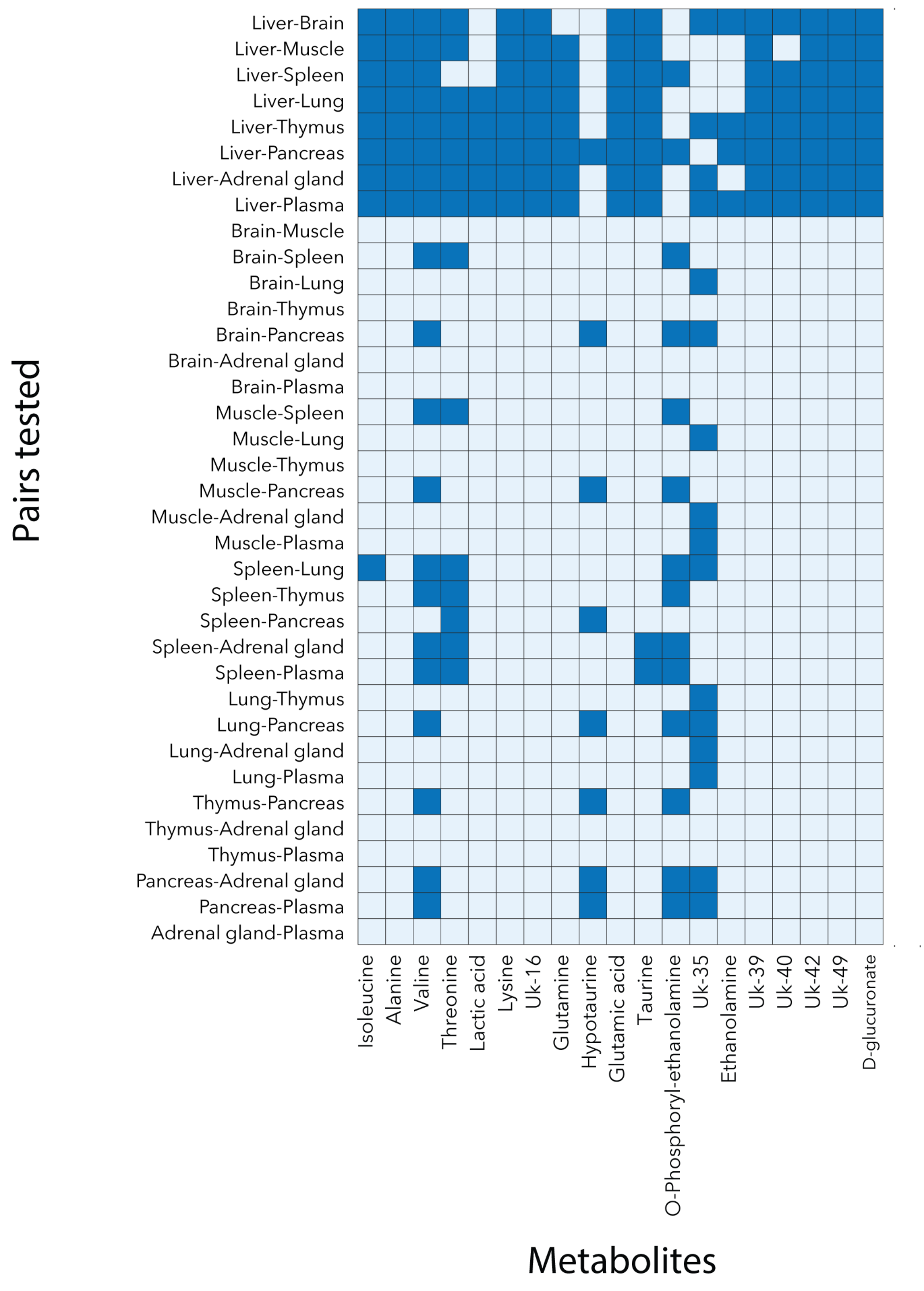

Supplementary Figure S3 Results of ANOVA and multiple comparison. Metabolites intensities were compared between tissues for the three mice. When there is a significant difference between tissues, they are highlighted in dark blue ($p < 0.05$ with Bonfferoni Correction). This information is summarized in Figure 3.

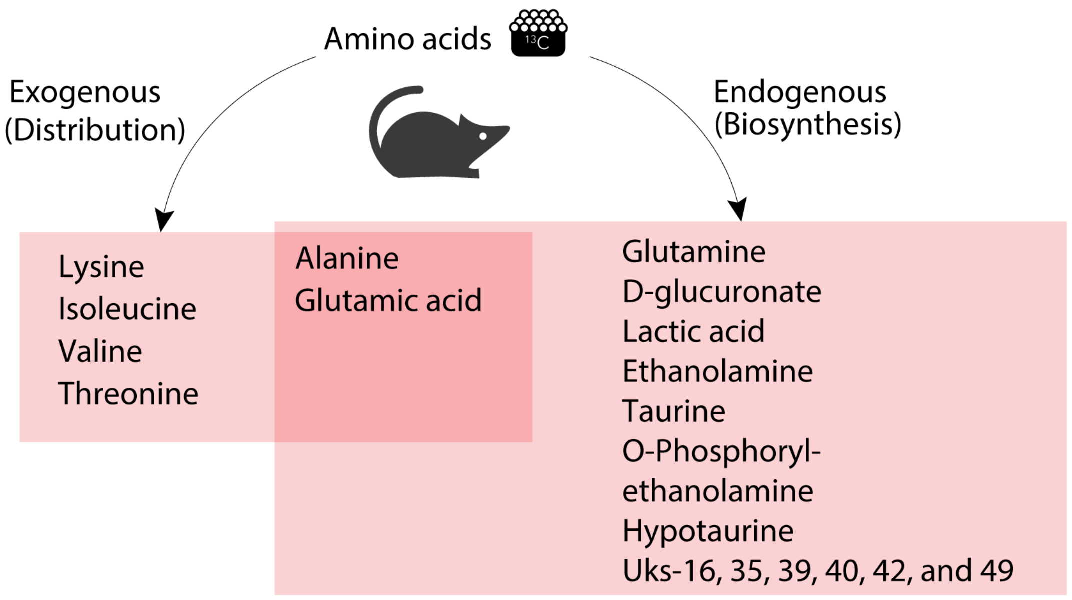

Supplementary Figure 4 Possible sources of metabolites in this study

References

1. Uchimiya, M.; Olofsson, M.; Powers, M. A.; Hopkinson, B. M.; Moran, M. A.; Edison, A. S., ^{13}C NMR metabolomics: J-resolved STOCSY meets INADEQUATE. *J Magn Reson* **2023,** *347*, 107365.
2. Delaglio, F.; Grzesiek, S.; Vuister, G. W.; Zhu, G.; Pfeifer, J.; Bax, A., NMRPipe - A multidimensional spectral processing system based on Unix pipes. *J Biomol Nmr* **1995,** *6* (3), 277-293.
3. Dieterle, F.; Ross, A.; Schlotterbeck, G.; Senn, H., Probabilistic quotient normalization as robust method to account for dilution of complex biological mixtures. Application in H-1 NMR metabonomics. *Anal Chem* **2006,** *78* (13), 4281-4290.
4. Dashti, H.; Westler, W. M.; Markley, J. L.; Eghbalnia, H. R., Unique identifiers for small molecules enable rigorous labeling of their atoms. *Sci Data* **2017,** *4*, 170073.
5. Clendinen, C. S.; Pasquel, C.; Ajredini, R.; Edison, A. S., ^{13}C NMR metabolomics: INADEQUATE network Analysis. *Anal Chem* **2015,** *87* (11), 5698-5706.
